# Prognostic Values and Clinical Significance of S100 Family Member’s Individualized mRNA Expression in Pancreatic Adenocarcinoma

**DOI:** 10.3389/fgene.2021.758725

**Published:** 2021-11-03

**Authors:** Xiaomin Li, Ning Qiu, Qijuan Li

**Affiliations:** ^1^ Guangzhou Women and Children’s Medical Center, Guangzhou Medical University, Guangzhou, China; ^2^ Southern Marine Science & Engineering Laboratory (Guangzhou), Guangzhou, China; ^3^ Department of Clinical Laboratory, The Fifth Affiliated Hospital of Sun Yat-sen University, Zhuhai, China

**Keywords:** mRNA expression, S100 family, pancreatic cancer, biomarker, prognosis

## Abstract

**Objective:** Pancreatic adenocarcinoma (PAAD) is a common malignant tumor worldwide. S100 family (S100s) is wildly involved in regulating the occurrence, development, invasion, metastasis, apoptosis, and drug resistance of many malignant tumors. However, the expression pattern, prognostic value, and oncological role of individual S100s members in PAAD need to be elucidated.

**Methods:** The transcriptional expression levels of S100s were analyzed through the Oncomine and GEPIA, respectively. The protein levels of S100s members in PAAD were studied by Human Protein Atlas. The correlation between S100 mRNA expression and overall survival and tumor stage in PAAD patients was studied by GEPIA. The transcriptional expression correlation and gene mutation rate of S100s members in PAAD patients were explored by cBioPortal. The co-expression networks of S100s are identified using STRING and Gene MANIA to predict their potential functions. The correlation of S100s expression and tumor-infiltrating immune cells was tested by TIMER. Pathway activity and drug target analyzed by GSCALite.

**Results:** 13 S100s members were upregulated in PAAD tissues. 15 S100s members were associated with TP53 mutation. Expression levels of S100A3/A5/A6/A10/A11/A14/A16/B/P/Z were significantly correlated with the pathological stage. Prognosis analysis demonstrated that PAAD patients with low mRNA levels of S100A1/B/Z or high levels of S100A2/A3/A5/A10/A11/A14/A16 had a poor prognosis. Immuno-infiltration analysis showed that the mRNA levels of S100A10/A11/A14/A16 were correlated with the infiltration degree of macrophages in PAAD. Drug sensitivity analysis showed that PAAD expressing high levels of S100A2/A6/A10/A11/A13/A14/A16 maybe resistant to small molecule drugs.

**Conclusion:** This study identifies the clinical significance and biological functions of the S100s in PAAD, which may provide novel insights for the selection of prognostic biomarkers.

## Introduction

Pancreatic adenocarcinoma (PAAD) is a highly lethal disease and has become the seventh leading cause of cancer-related deaths, accounting for about 4.7% of global mortality (Sung et al., 2021). PAAD is associated with a very poor prognosis, with a 5-years survival rate of as low as 8% after diagnosis (Mishra et al., 2019). The low survival rate is attributed to various factors, which is mainly caused by the high rate of advanced PAAD since over 50% of PAAD patients are diagnosed at an advanced stage (Ilic and Ilic, 2016). Moreover, PAAD is characterized not only by early recurrence and invasion but also the resistance to chemotherapy and radiotherapy (Adamska et al., 2018). Although great strides have been made on the screening, diagnosis and comprehensive therapy combining surgery, chemotherapy, and radiotherapy, PAAD remains a highly malignant tumor with limited treatment options (Kamisawa et al., 2016). Conventional treatments, such as surgery, have poor clinical outcomes, and only about 20% of patients benefiting from radical surgery (Lai et al., 2019). At present, there are no reported cases of good efficacy of targeted therapy for pancreatic cancer driver genes (Zhang et al., 2021). The prognosis of PAAD is largely determined by early diagnosis and treatment. Therefore, seeking for key genes and proteins related to the occurrence, development, and metastasis of PAAD is of great significance in improving the prognosis.

Invasion and metastasis are typical events during the malignant progression of tumors, alongside tumor cell proliferation, shedding, dissemination, angiogenesis, implantation, and other aspects (Marchesi et al., 2010). Various proteins found to be implicated in tumorigenesis and tumor development. Calcium-binding proteins are a large family, which are responsible for mediating the cell cycle progression, cell differentiation, enzyme activation, muscle contraction, etc. (Donato, 1999). The S100 family (S100s) is one of the largest subfamilies of calcium-binding proteins, which plays a key role in cell proliferation, apoptosis, differentiation, and inflammation. So far, at least 20 members (S100A1-A14/A7A/A16/B/P/G/Z, etc) of the S100s have been reported (Allgöwer et al., 2020a). However, these members have some commonalities and differences in their respective organizational structures, which may make them play different roles in the occurrence and development of tumors. The chromosomal regions encoding S100s genes have poor stability and they are closely associated with the occurrence and development of tumors. Once tumorigenesis initiates, the gene in this region is easy to recombine and interfere with the S100s gene (Engelkamp et al., 1993; Schäfer et al., 1995; Marenholz et al., 2004; Marenholz et al., 2006; Goh et al., 2017). Thus, S100s are usually dysregulated in human malignant tumors, including PAAD (Bresnick et al., 2015; Xue et al., 2015). S100s have distinct expression and function patterns in tumorigenesis and tumor development. For example, S100A4/A7/A8/A9/A13 was found to have tumorigenesis role, however, S100A6/A8/A9 found to have anti-tumor activity (Salama et al., 2008). S100s members can also participate in the regulation of various biological functions associated with the progress of PAAD. The expression of S100P/A4 have been proven to be related to the differentiation, metastasis, prognosis, and drug resistance of pancreatic cancer. S100A2/A10 have recently been suggested as negative prognostic biomarkers for pancreatic cancer (Bachet et al., 2013; Bydoun et al., 2018a). S100P/A11 are unfavorable to the prognosis of PAAD patients undergoing surgical resection (Xiao et al., 2012; Camara et al., 2020). Collectively, the clinical significance of S100s members and their potential application in the development of PAAD have been highlighted, although their predictive potential and biological characteristics need to be further validated. Moreover, the relationship between S100s and immune cell infiltration and drug resistance of PAAD remains unclear (Chen et al., 2021).

In our study, with the help of GEPIA, we systematically evaluated the transcriptional expressions of the S100s and their relationship with tumor stage and prognostic signature in PAAD. Based on data mining and analyses, we also clarified the gene mutation, potential biological roles, and drug resistance of S100s members in PAAD. Besides, we also assessed the correlation between S100s mRNA expression and immune cell infiltration in PAAD using the tool TIMER.

## Materials and Methods

### Oncomine

Oncomine gene expression array database (Adamska et al., 2018; Ma et al., 2019) was used to analyze the mRNA level of S100s members in various cancers. The student’s t-test was used to generate a *p*-value for the comparison between clinical cancer specimens and pair control tissues. The thresholds for each S100s member were set at fold change:2; *p*-value: 0.001; gene rank: 10%.

### Gene expression profile Interactive Analysis

Gene expression profile Interactive Analysis (GEPIA) is a bioinformatics analysis tool for evaluating RNA expression, which contains 9,736 tumors and 8,587 normal samples from the Cancer Genome Atlas and Genotype-tissue Expression dataset (Tang et al., 2017). The database delivers rapid and customizable functionalities, including differential expression analysis, profiling plotting, correlation analysis, patient survival analysis, similar gene detection, and dimensionality reduction analysis.

### cBioPortal

CBioPortal is a bioinformatics analysis tool, providing a comprehensive analysis of complex cancer genomics and clinical characteristics (Gao et al., 2013). The pancreatic adenocarcinoma (TCGA, Firehose Legacy) dataset including data from 186 cases with pathology reports was chosen for further analyses of the S100s. We used it to analyze the genetic mutation, co-expression, and pathway of S100s.

### STRING and GeneMANIA

STRING is a comprehensive publicly available bioinformatics database, providing network prediction of protein-protein interactions based on physical and functional correlations (Szklarczyk et al., 2019). We used it to construct the S100s interaction relationship network and explore the interaction among the S100s to predict the core proteins and key candidate genes. GeneMANIA is a flexible prediction platform, which can construct gene interaction networks by predicting co-expression, physical interactions, interactions, shared protein domains, and pathways (Warde-Farley et al., 2010). We use it to establish S100s gene co-expression networks to predict their potential function.

### TIMER

TIMER is a useful and flexible web interface, providing six main analysis modules to systematically evaluate the infiltration and clinical effects of distinct immune populations in the tumor microenvironment (Li et al., 2020a). We used TCGA_PAAD datasets to evaluate the correlation between S100s expression and the abundance of immune infiltrating cells by Spearman correlation analysis.

### GSCALite

GSCALite is a comprehensive publicly available bioinformatics database for genomic set cancer analysis, including expression, single nucleotide variation (SNV), copy number variation (CNV), methylation, small molecular drug targets, and cancer pathway activity analysis. The pathway activity module represents the correlation between gene expression and the pathway activity group (inhibition and activation) determined by the pathway score. Genome aberration not only affects the clinical therapeutic response but also provides a large number of research targets for the study of potential drug targets (Liu et al., 2018). GSCALite database integrates the drug sensitivity and gene expression profile data of cancer cells in GDSC and CTRP. Researchers can use this database to mine potential biomarkers and predict valuable small drugs, which is conducive to better research design and clinical trials in the future. We used it to analyze the pathway activity and drug targets of the S100s.

### Human Protein Atlas

The Human Protein Atlas (HPA) is a valuable platform for studying the localization and expression of proteins, as it contains more than 10 million immunohistochemistry images and 82,000 high-resolution immunofluorescence images (Thul and Lindskog, 2018). The protein levels of S100s members in normal and PAAD pancreatic tissues were compared using representative immunohistochemical images of HPA.

### UALCAN

UALCAN is a user-friendly online website for analyzing cancer OMICS data (TCGA, MET500, and CPTAC) (Sighoko et al., 2011). We used it to analyze the relationships between S100s mRNA expression and TP53 mutation.

## Results

### Expression Level of the S100s Members in Pancreatic Adenocarcinoma

**FIGURE 1:**
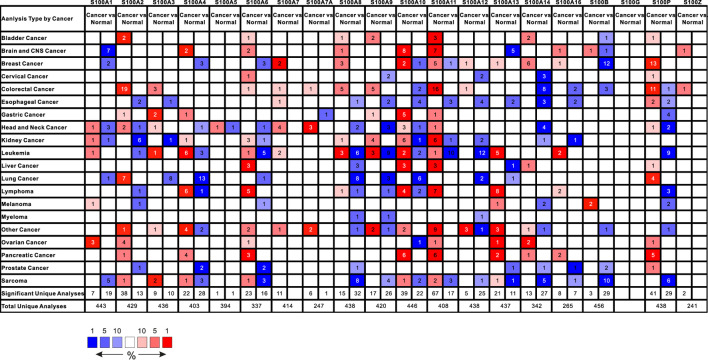
The mRNA expression of the S100 family members (S100s) in 20 different cancer types using ONCOMINE. Color was determined by the best gene rank percentile gene for analyses within the cell; blue represented down-expression and red represented over-expression. The numbers in colored cells represents the quantities of datasets which satisfies the threshold: gene rank percentile (10%), P-value (0.001), and fold change (2.0). The mRNA expression of S100A2/A4/A6/A10/A11/A13/14/A16/P were higher in tumor than in normal pancreatic tissues.

Transcriptional levels of the S100s members between tumor and normal samples in twenty types of cancers were assessed by the tool ONCOMINE. As shown in Figure 1, nine S100s members, including S100A2/A4/A6/A10/A11/A13/A14/A16/P were significantly upregulated in pancreatic cancer (fold change = 2, *p* < 0.001). Then, through the oncology database, we further studied the significant differences in S100s transcription levels between distinct subtypes of pancreatic cancer and normal control tissues, as demonstrated in Table 1. In Pei’s dataset, the mRNA expression level of S100A2 was overexpressed in pancreatic carcinoma versus normal samples with a fold change of 7.68 (Table 1). In Logsdon’s datasets, S100A4 was found to be higher expressed in pancreatitis (fold change = 2.57), and pancreatic adenocarcinoma (fold change = 4.44) versus normal tissues. Badea et al. reported that S100A4 was increased in pancreatic ductal adenocarcinoma (fold change = 4.37), and Pei et al. showed that S100A4 also overexpressed in Pancreatic carcinoma (fold change = 4.86) compared to normal samples. In Badea’s dataset, S100A6 was found higher expressed in pancreatic ductal adenocarcinoma compared to normal tissues (fold change = 5.92). Overexpression of S100A6 in pancreatic carcinoma was also found in Pei’s dataset (fold change = 9.15) and Segara’s dataset (fold change = 4.76). Additionally, in Logsdon’s datasets, S100A10/A11 were overexpressed in pancreatic adenocarcinoma (fold change = 7.58 and 18.29), and pancreatitis (fold change = 2.97 and 5.45) compared to normal samples. According to Segara’s dataset, S100A10/A11 were also found higher expressed in pancreatic carcinoma (fold change = 4.3 and 7.52). In Badea’s datasets, higher expressed S100A10/A11 were found in pancreatic ductal adenocarcinoma ((fold change = 3.1 and 4.43). In Iacobuzio-Donahue’s dataset, S100A10/A11 were showed overexpressed in pancreatic adenocarcinoma (fold change = 5.54 and 7.20). Additionally, in Pei’s datasets, S100A10/A11/A14/A16/P were found higher expressed in pancreatic carcinoma (fold change = 3.54, 4.95, 6.46, 4.40, 77.93) in comparison with normal samples. S100A13/P were significantly upregulated in pancreatic carcinoma, with fold changes of 2.68 and 17.73 in Segara’s dataset. Higher expressed S100A13/16/P were found in pancreatic ductal adenocarcinoma (fold change = 2.19, 2.33, 13.18) of Badea’s datasets. S100P in pancreatic adenocarcinoma showed a similar trend, with 24.02 and 20.3-fold changes in Iacobuzio-Donahue’s and Logsdon’s datasets, respectively. According to ONCOMINE analysis, there was no significant difference in mRNA expression of S100A1/A3/A5/A7/A7A/A8/A9/A12/B/G/Z between patients with PAAD and normal controls.

**TABLE 1:** Significant changes in the transcriptional expression of the S100 gene family members between PAAD and pancreatic normal samples (OnCOMine database).

Gene	Type of pancreatic cancer vs normal samples	Fold change	t-test	*P* Value	Reference	Tumor samples	Normal samples
S100A2	Pancreatic carcinoma vs normal	7.68	6.38	2.97E-08	Pei	36	16
S100A4	Pancreatitis vs normal	2.57	5.70	4.45E-04	Logsdon	10	5
	Pancreatic adenocarcinoma vs normal	4.44	5.74	6.52E-05	Logsdon	10	5
	Pancreatic ductal adenocarcinoma vs normal	4.37	7.61	1.54E-10	Badea	36	36
	Pancreatic carcinoma vs normal	4.86	5.57	3.49E-06	Pei	36	16
S100A6	Pancreatic carcinoma vs normal	9.15	9.15	4.98E-12	Pei	36	16
	Pancreatic ductal adenocarcinoma vs normal	5.92	9.02	1.32E-12	Badea	36	36
	Pancreatic carcinoma vs normal	4.76	4.84	2.31E-04	Segara	12	6
S100A10	Pancreatic carcinoma vs normal	4.30	8.15	8.32E-07	Segara	12	6
	Pancreatic adenocarcinoma vs normal	7.58	10.08	2.79E-06	Logsdon	10	5
	Pancreatitis vs normal	2.97	5.13	4.89E-04	Logsdon	10	5
	Pancreatic ductal adenocarcinoma vs normal	3.10	8.38	1.31E-11	Badea	36	36
	Pancreatic adenocarcinoma vs normal	5.54	6.07	7.36E-04	Iacobuzio-Donahue	17	5
	Pancreatic carcinoma vs normal	3.54	6.06	2.05E-06	Pei	36	16
S100A11	Pancreatic carcinoma vs normal	7.52	7.65	7.48E-07	Segara	12	6
	Pancreatic ductal adenocarcinoma vs normal	4.43	10.45	1.19E-14	Badea	36	36
	Pancreatitis vs normal	5.45	5.51	7.60E-04	Logsdon	10	5
	Pancreatic adenocarcinoma vs normal	18.29	10.08	9.25E-05	Logsdon	10	5
	Pancreatic adenocarcinoma vs normal	7.20	6.01	8.54E-04	Iacobuzio-Donahue	17	5
	Pancreatic carcinoma vs normal	4.95	5.83	6.35E-06	Pei	36	16
S100A13	Pancreatic carcinoma vs normal	2.68	6.00	1.85E-05	Segara	12	6
	Pancreatic ductal adenocarcinoma vs normal	2.19	7.26	3.32E-10	Badea	36	36
S100A14	Pancreatic carcinoma vs normal	6.46	6.50	4.85E-07	Pei	36	16
S100A16	Pancreatic Carcinoma vs Normal	4.40	7.26	9.46E-08	Pei	36	16
	Pancreatic Ductal Adenocarcinoma vs Normal	2.33	6.94	1.05E-09	Badea	36	36
S100P	Pancreatic Adenocarcinoma vs Normal	24.02	11.30	2.13E-08	Iacobuzio-Donahue	17	5
	Pancreatic Adenocarcinoma vs Normal	20.31	15.50	4.88E-10	Logsdon	10	5
	Pancreatic Carcinoma vs Normal	77.93	10.19	1.61E-12	Pei	36	16
	Pancreatic Carcinoma vs Normal	17.73	6.94	1.23E-05	Segara	12	6
	Pancreatic Ductal Adenocarcinoma vs Normal	13.18	8.44	8.53E-13	Badea	36	36

**FIGURE 2:**
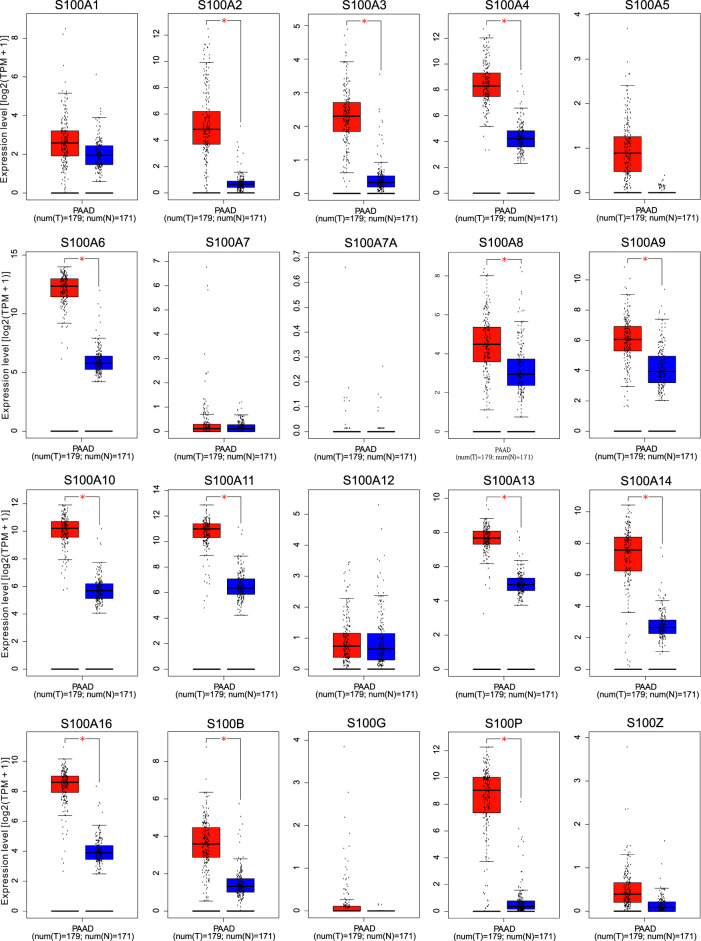
The transcription levels of the S100 family members in Pancreatic adenocarcinoma patients using GEPIA. Box plots from GEPIA gene expression data compared the expression of specific S100 family members in PAAD and normal tissues. Select log2 (TPM + 1) transformed expression data for plotting. Blue color represents normal tissue and red color represents tumor tissue. The levels of S100A2/A3/A4/A6/A8/A9/A10/A11/A13/A14/A16/B/P in PAAD tissues were higher than that in normal paired samples as determined by Student’s t-test. *p* < 0.05 was considered to be statistically significant. **Abbreviation:** num, sample number; T, tumor; N, normal; TPM, Transcripts per million. * Demonstrate that the results were statistically significant.

Next, we utilized the Gene Expression Profiling Interactive (GEPIA) dataset to confirm the mRNA expression levels of differentially expressed S100s factors in PAAD and corresponding control tissues shown in the Oncomine database. We found that the transcription levels of S100A2/A3/A4/A6/A8/A9/A10/A11/A13/A14/A16/B/P were all highly expressed in PAAD tissues as compared with normal pancreatic tissues. Other S100 gene family members including S100A1/A5/A7/A7A/A12/G/Z have indicated no significant differences between PAAD and normal tissues (Figure 2 and Supplementary Figure S1).

**FIGURE 3:**
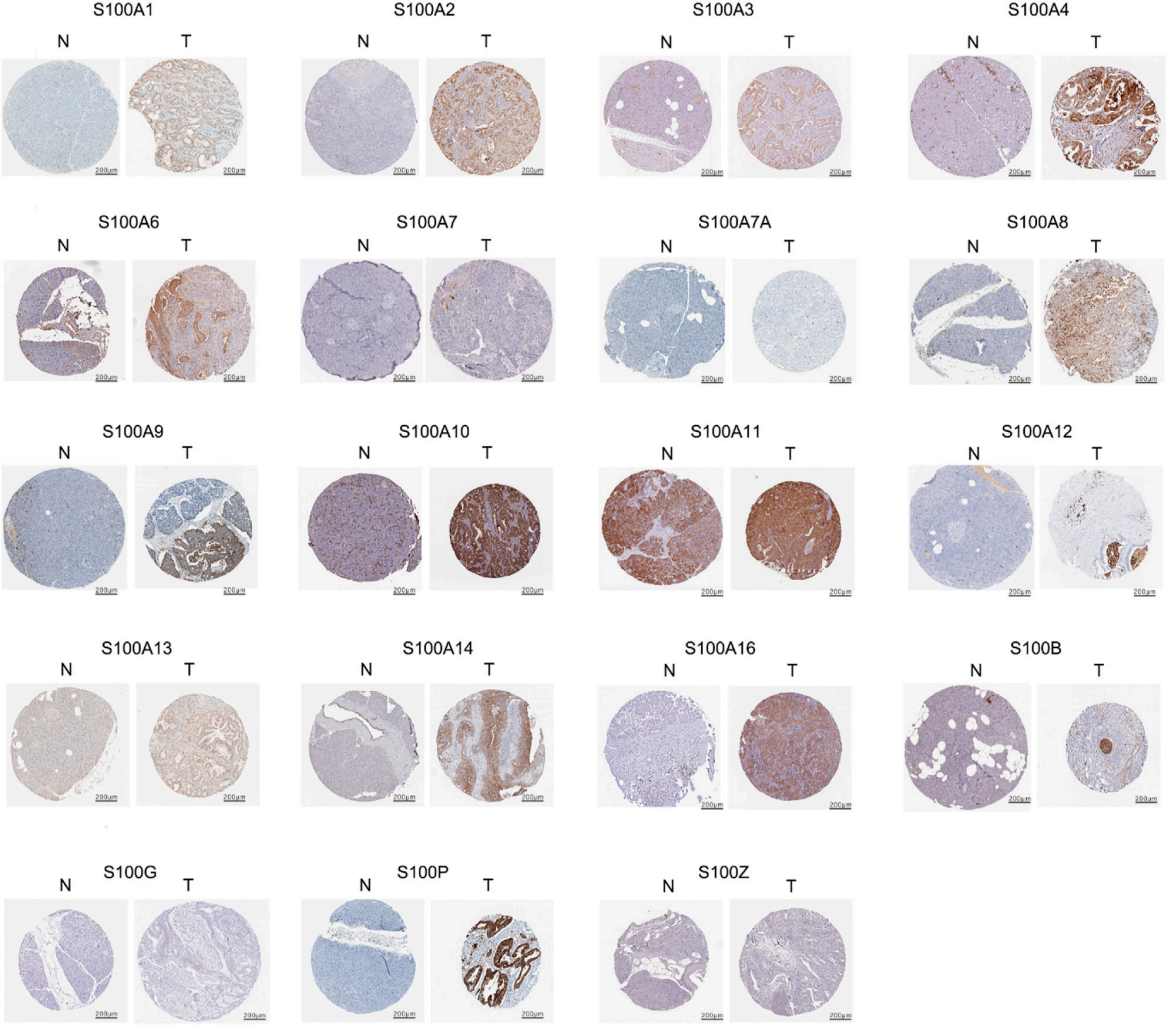
Representative immunohistochemistry images (IHC) of the S100 family members in PAAD and normal pancreatic tissues using HPA database. The expression of S100A5 protein was not shown in the database. The expression of S00A7/A7A/A8/A9/A12/A14/A16/B/P/G/Z proteins were not detected in normal pancreatic tissues. The remain of S100 protein was expressed at low to moderate levels in some normal pancreatic tissues. Low protein expression of S100A2/A3/A7/A7A/A8/A13 were detected in PAAD tissues. Medium protein expression of S100A1/A4/A6/A9/A11/A16/B were detected in PAAD tissues. High protein expression of S100A10/A14/P were observed in PAAD tissues.

After detecting the transcriptional level of the S100s in pancreatic adenocarcinoma tissues, we used the HPA to study its protein expression level. As shown in Figure 3, the expression of S00A7/A7A/A8/A9/A12/A14/A16/B/P/G/Z protein was not detected in normal pancreatic tissues, while the remaining S100 protein was expressed at low to moderate levels in some normal pancreatic tissues. The expression of S100A5 protein was not shown in the database. Low expression (protein expression scored <25%) of S100A2/A3/A7/A7A/A8/A13 protein was detected in PAAD tissues. Medium expression (protein expression scored ranged from 25 to 75%) of S100A1/A4/A6/A9/A11/A16/B protein was detected in PAAD samples. High expression (protein expression scored >75%) of S100A10/A14/P protein was observed in PAAD tissues. However, due to the small size of the PAAD immunohistochemical results in the HPA database, the conclusion remains to be further verified.

### The Relationship Between the mRNA Expression Levels of the S100s Members and Clinical Characteristics in Pancreatic Adenocarcinoma

**FIGURE 4:**
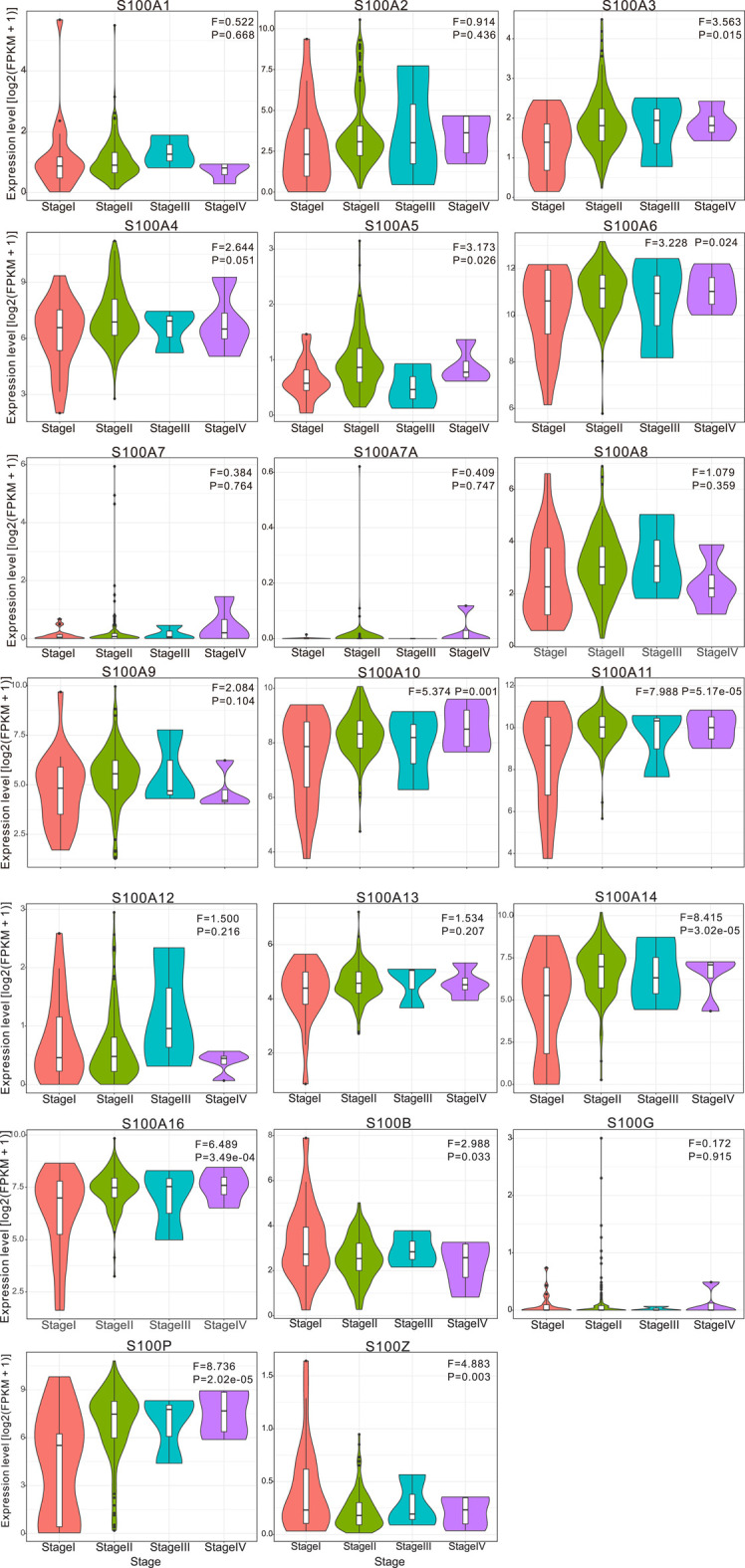
Violin plot demonstrated the correlation between S100s transcription level and tumor stages in patients with PAAD (calculated data form TCGA-PAAD). The difference of individual S100s gene expression in each stage was analyzed by one-way ANOVA, in which Pr (>F) < 0.05 was considered to be statistically significant. The number of pancreatic cancer samples at each tumor stage was 21 cases in stage I, 146 in stage II, 3 in stage III, and 4 in stage IV. **Abbreviation:** F value, the statistical value of F test; Pr (>F), *p*-value.

To evaluate the clinical significance of the differentially expressed S100 gene in the progression of PAAD patients, we analyzed the correlation between the transcriptional expression level of S100s members and clinicopathological features. The original data we used were derived from TCGA databases. We found that the transcriptional levels of S100A3/A5/A6/A10/A11/A14/A16/B/P/Z were correlated with tumor stage (Figure 4). There was no significant correlation between the remaining S100s members and the tumor stage.

**FIGURE 5:**
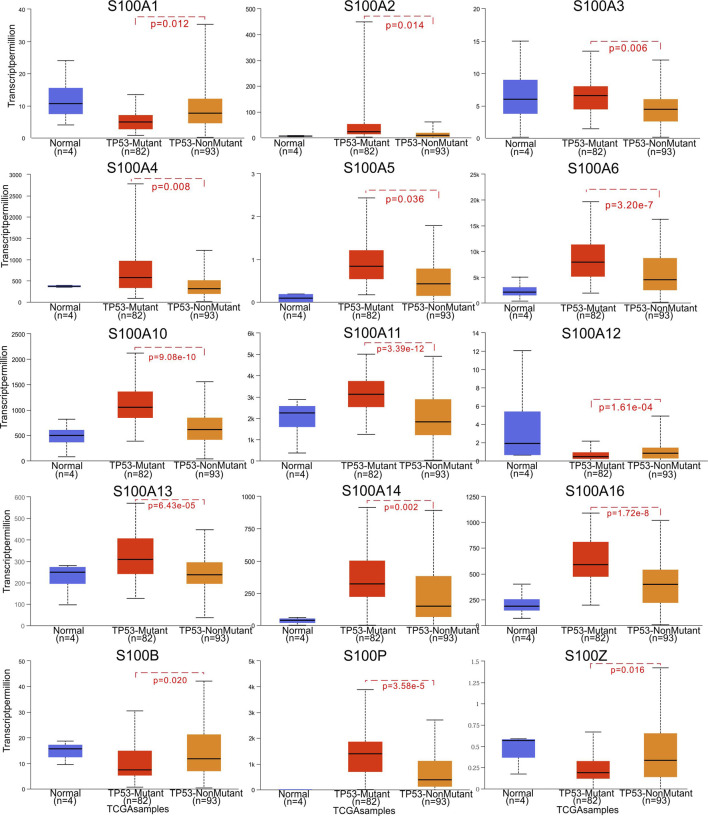
Correlation between S100s mRNA expression and TP53 mutation in PAAD (UALCAN). The significance of difference between TP53-mutant and TP53-nonmutant estimated by Student’s *t*-test with p value. The sample number N and the Student’s *t*-test p value are marked on the figure, respectively.

We then explored the prognostic value of S100 family members in pancreatic cancer of different TP53 status. We found that the high expression of S100A2/A3/A4/A5/A6/A10/A11/A13/A14/A16/P in pancreatic cancer was positively correlated with TP53 mutation, while the high expression of S100A1/A12/B/Z was negatively correlated with TP53 mutation (Figure 5).

### Prognostic Values of S100s in Patients With Pancreatic Adenocarcinoma

**FIGURE 6:**
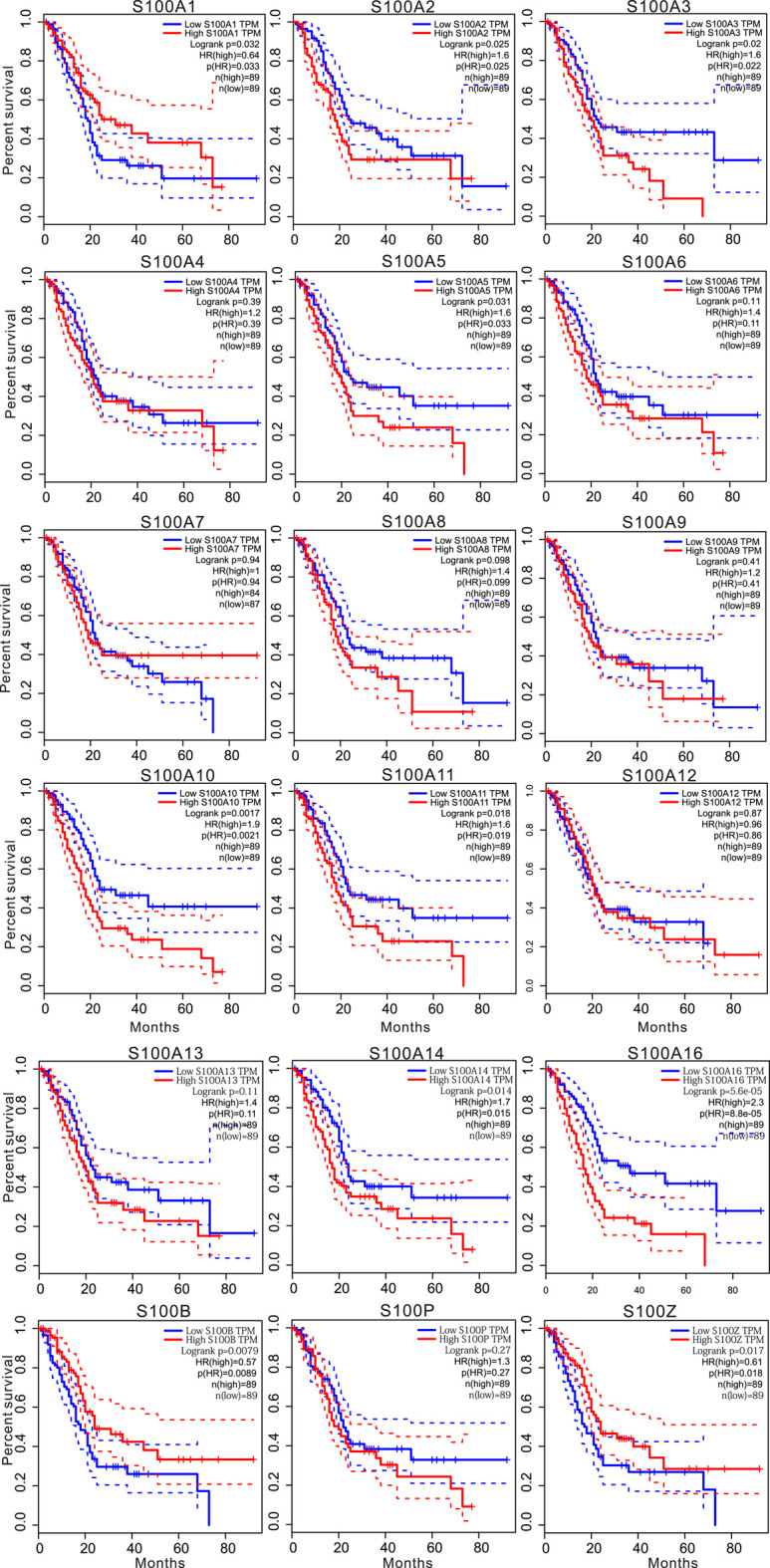
Kaplan-Meier survival analysis showed that higher mRNA expression of S100A2/A3/A5/A10/A11/A14/A16 was significantly associated with shorter survival time, while higher expression of S100A1/B/Z was associated with longer survival time (GEPIA,n = 178). The *p-*value was less than 0.05.

To further explore the key role of S100s members in the survival of PAAD, we used the GEPIA database to analyze the relationship between the expression of S100 factors and the overall survival (OS) of patients with PAAD. As displayed in Figure 6, we found that the high transcriptional levels of S100A2 (Hazard ratio (HR) = 1.6, *p* = 0.025), S100A3 (HR = 1.6, *p* = 0.02), S100A5 (HR = 1.6, *p* = 0.031), S100A10 (HR = 1.9, *p* = 0.0017), S100A11 (HR = 1.6, *p* = 0.018), S100A14 (HR = 1.7, *p* = 0.014) and S100A16 (HR = 2.3, *p* = 5.6e-05) were significantly associated with poor OS in PAAD, while low mRNA expression of S100A1 (HR = 0.64, *p* = 0.032), S100B (HR = 0.57, *p* = 0.0079) and S100Z (HR = 0.61, *p* = 0.017) were associated with worse OS. However, the statistical significance of S100A4 (HR = 1.2, *p* = 0.39), S100A6 (HR = 1.4, *p* = 0.11), S100A7 (HR = 1, *p* = 0.94), S100A8 (HR = 1.4, *p* = 0.098), S100A9 (HR = 1.2, *p* = 0.41), S100A12 (HR = 0.96, *p* = 0.87), S100A13 (HR = 1.4, *p* = 0.11), S100P (HR = 1.3, *p* = 0.27) was not detected. The GEPIA databases did not provide the survival analysis results of S100A7A and S100G in the majority of PAAD molecular subtypes. Thus, the PAAD patients with low mRNA levels of the S100A1/B/Z or high mRNA levels of S100A2/A3/A5/A10/A11/A14/A16 were predicted to have worse OS.

### Genetic Mutation and Interaction Networks Analysis of S100s Members in Pancreatic Adenocarcinoma

**FIGURE 7:**
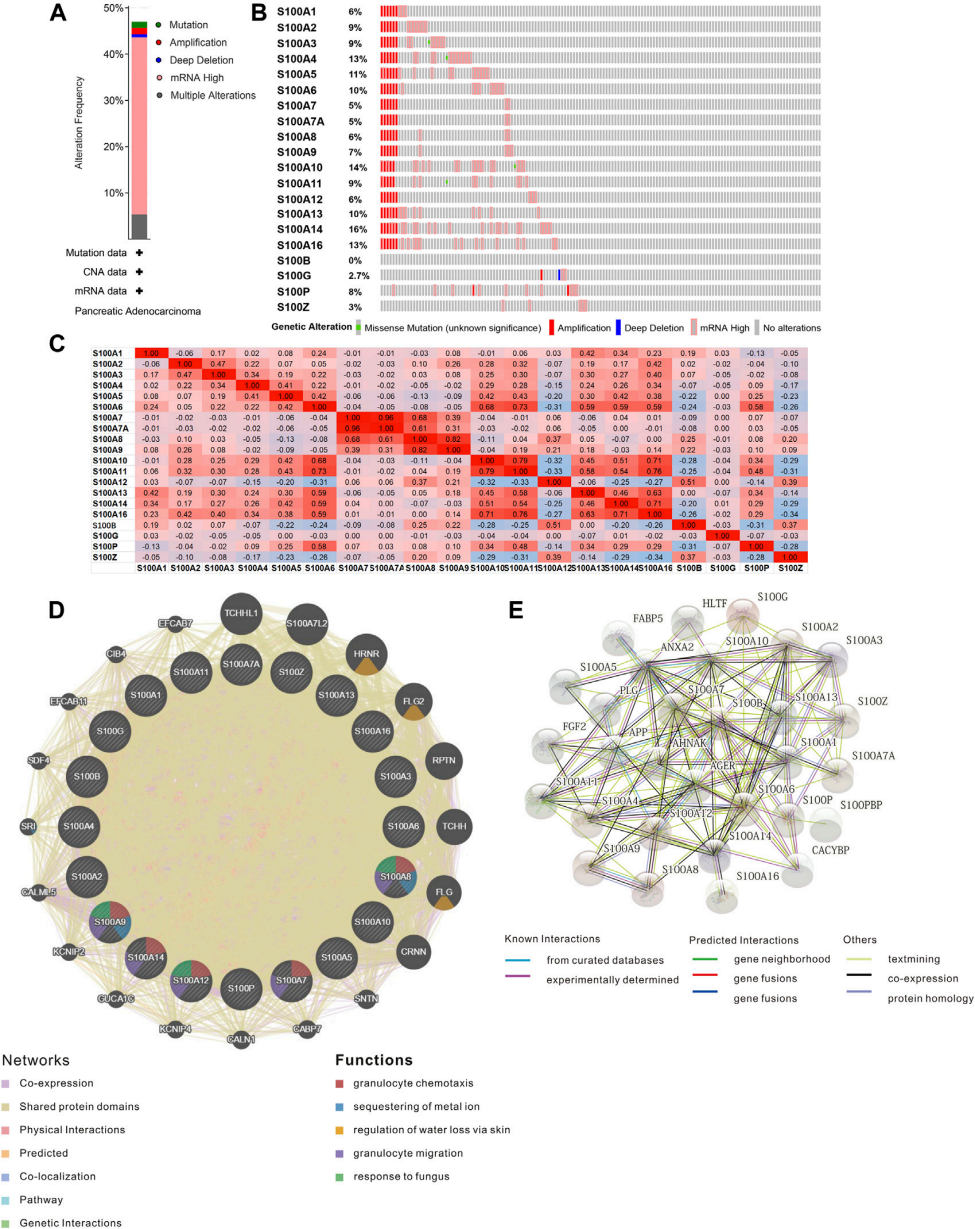
Genetic mutation and interaction networks analysis of the S100s members in PAAD (cBioPortal, GeneMINIA, and STRING). **(A)** Summary of mutation frequency in S100 factors in PAAD (n=149), **(B)** Genetic alteration of each S100s in PAAD (n=149), **(C)** Correlation among different S100s factors in PAAD by Pearson's correlation coefficients based on cBioportal databases (positive correlation is red, negative correlation is blue, n=149), **(D)** Gene interaction networks among S100s in the GeneMANIA database. **(E)** Protein-protein interaction network of the S100s members in PAAD.

The S100 alterations and correlations were analyzed by the tool cBioPortal for Pancreatic Adenocarcinoma (TCGA, Firehose Legacy). S100s were altered in 70 samples of 149 patients with PAAD, accounting for 47% (Figure 7A). As shown in Figure 7B, the genetic alteration rates of the S100 gene family members in PAAD ranges from 0 to 16% individually (S100A1, 6%; S100A2, 9%; S100A3, 9%; S100A4, 13%; S100A5, 11%; S100A6, 10%; S100A7, 5%; S100A7A, 5%; S100A8, 6%; S100A9, 7%; S100A10, 14%; S100A11, 9%; S100A12, 6%; S100A13, 10%; S100A14, 16%; S100A16, 13%; S100B, 0%; S100G, 2.7%; S100P, 8%; S100Z, 3%).

Meanwhile, the transcriptional expression correlation of S100s members individually in patients with PAAD (TCGA, Firehose Legacy) was calculated by the cBioPortal database, and Pearson correlation analysis was carried out. The statistical significance is as follows: S100A6 with S100A10/A11/A13/A14/A16/P; S100A7 with S100A7A/A8; S100A7A with S100A8; S100A8 with S100A9; S100A10 with S100A11/A14/A16; S100A11 with S100A13/A14/A16; S100A12 with S100A13; S100A13 with S100A16; S100A14 with S100A16 (Figure 7C).

The interaction relationship and potential regulatory mechanism of S100s factors in PAAD were mined by using the GeneMANIA database, and the gene interaction network was constructed. The network consists of 40 genes, including 20 members of the S100s and another 20 genes extracted from the GeneMANIA. The analysis illustrated that there was a close genetic relationship among the members of the S100s. The results displayed that the S100s was co-expressed interactively with TCHHL1, S100A7L2, HRNR, FLG2, RPTN, TCHH, FLG, CRNN, SNTN, CABP7, CALN1, KCNIP4, GUCA1C, KCNIP2, CALML5, SRI, SDF4, EFCAB11, CIB4, EFCAB7 (Figure 7D). The functions of the S100s were mainly associated with granulocyte chemotaxis, sequestering of metal ions, regulation of water loss via the skin, granulocyte migration, and response to fungus (Figure 7D).

We used the tool STRING to construct a protein-protein interaction network of the S100s to explore their potential interactions. The networks contained 30 nodes and 122 edges (Figure 7E). The top five proteins most associated with S100s were ANXA2, AGER, AHNAK, HLTF, and S100PBP. The main biological processes involved in the PPI network were positive regulation of response to external stimulus, regulated exocytosis, astrocyte differentiation, myeloid leukocyte activation, and cell activation involved in the immune response. An IL-17 signaling pathway is the main pathway of KEGG.

### Cancer Pathway Activity and Drug Sensitivity Analysis of the S100s in Pancreatic Adenocarcinoma

**FIGURE 8:**
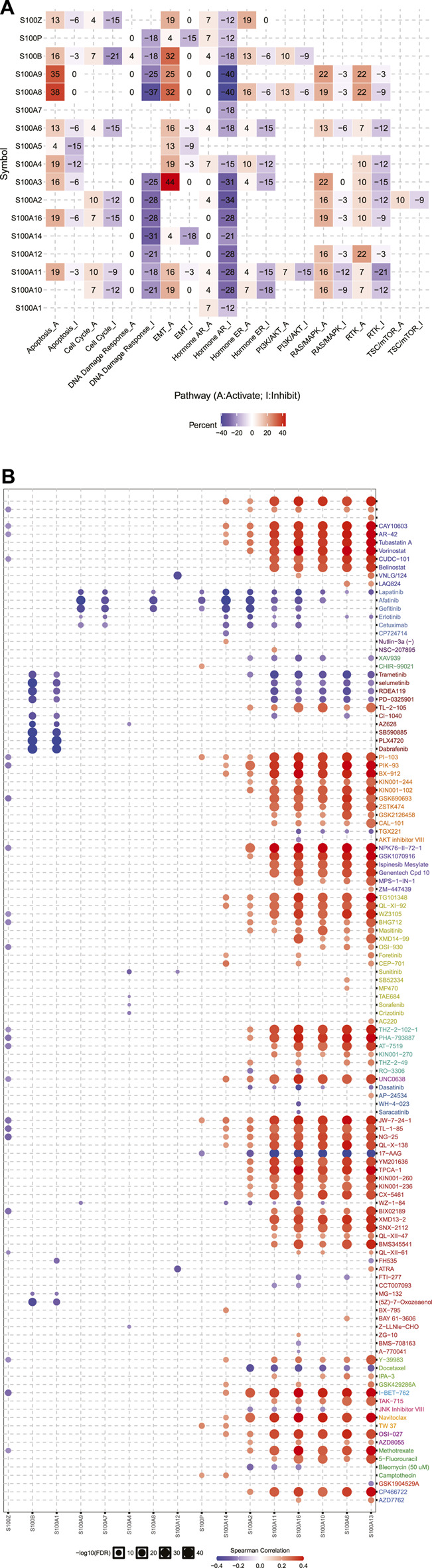
Cancer pathway activity and drug sensitivity analysis of the S100s in PAAD (GSCALite, n=178). **(A)** The role of the S100s in the famous cancer-related pathways. **(B)** Spearman correlation showed that S100 gene expression was related to small drugs. Negative correlation means that high gene expression is sensitive to the drug, and vice versa.

We further analyzed the role of the S100s in canonical cancer-related pathways using GSCALite and Cbioportal databases. Firstly, we used the platform GSCALite to analyze the activities of ten well-known tumor-related pathways, such as RTK, RAS/MAPK, TSC/mTOR, cell cycle, DNA damage response, EMT, hormone ER, hormone AR, PI3K/AKT, and apoptosis pathway. We found that most members of the S100s were associated with the activation of EMT, apoptosis, RTK, and RAS/MAPK pathway; and the inhibition of Hormone AR, DNA Damage Response, and RTK pathway (Figure 8A). Furthermore, we also analyzed the cancer pathway of the S100s based on cBioPortal. Through the analysis of the proportion of gene changes in ten typical carcinogenic signaling pathways, we found that the S100 alterations were closely related to the carcinogenic changes of key gene loci such as KRAS, CDKN2A, TP53, MYC, SMAD4 (with more than 10% mutation) (Supplementary Figure S2).

Gene mutations may affect clinical therapeutic responses and become potential drug targets. GSCALite was an publicly available platform that integrated the drug sensitivity and gene expression profile data of cancer cell lines in GDSC and CTRP, which was helpful for researchers to mine and predict valuable small drugs. Drug sensitivity indicated that the high expression of S100A2/A6/A10/A11/A13/A14/A16 was resistant to small molecule drugs or high expression of S100A1/A7/A9/B/Z were sensitive to small molecule drugs (Figure 8B).

### Correlation Between S100s and Immune Cell Infiltration in Patients With Pancreatic Adenocarcinoma

**FIGURE 9:**
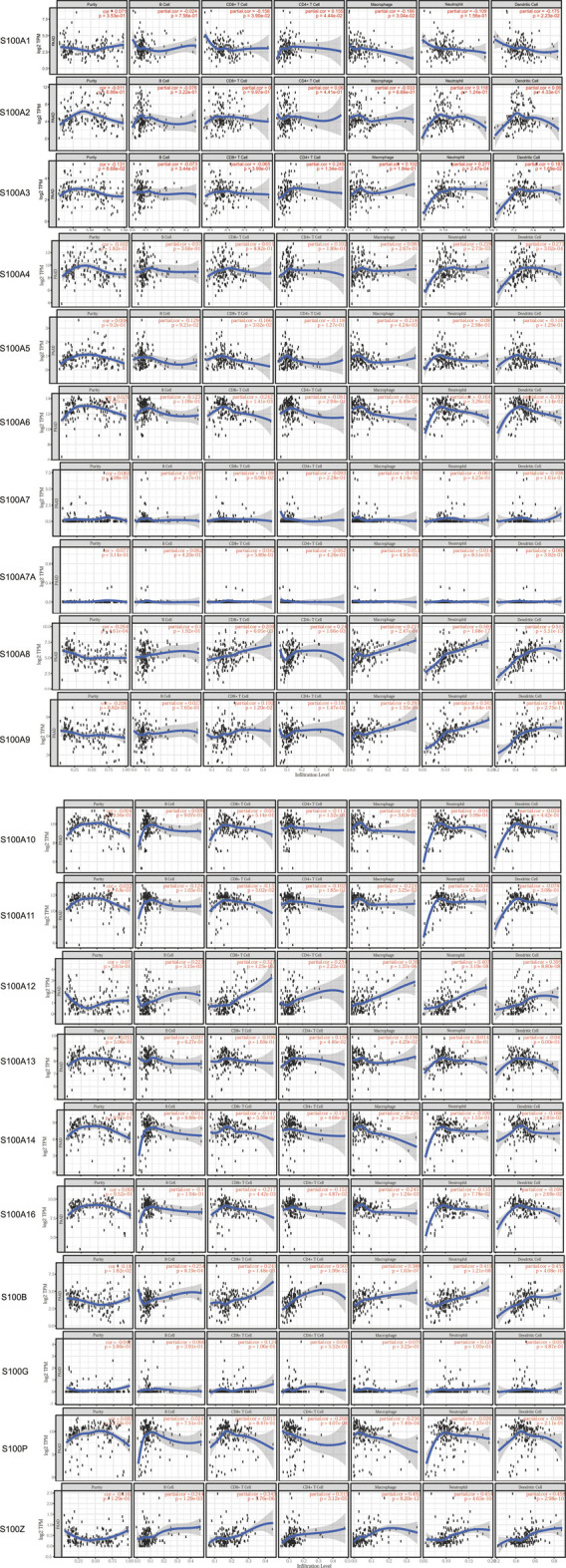
Correlations between S100 gene mRNA expression and immune infiltration abundance in PAAD (TIMER database, n=179). The mRNA expression of some S100s factors was correlated with the infiltration abundance of B cells, CD8+ T cells, CD4+ T cells, macrophages, neutrophils and dendritic cells.

In recent years, studies have shown that the enrichment of immune cells in the tumor microenvironment is closely related to tumor proliferation and development (de Visser et al., 2006; Lei et al., 2020). To evaluate the effect of the S100s on the degree of immune cell infiltration in the tumor microenvironment, we used TIMER to analyze the correlation between the expression level of the S100s members and immune infiltrating cells in PAAD. As shown in Figure 9, S100A3 was positively correlated with the abundance of CD4^+^ T cells, neutrophils, and dendritic cells. S100A4 showed a significant correlation with the abundance of neutrophils, and dendritic cells. S100A5 was negatively correlated with the infiltration of CD8^+^ T cells and macrophages. S100A14 was also negatively associated with the infiltration of CD4+cells, macrophage, and dendritic cell. Except for B cells and CD4^+^ T cells, S100A6 was negatively correlated with the abundance of the other immune cells (CD8^+^ T cells, macrophages, neutrophils, and dendritic cells. For S100A7/A10/A11, the expression of these genes was negatively associated with the abundance of macrophages. Meanwhile, the expression of S100A13/P was associated with the infiltration of CD4^+^ T cells and macrophages. Besides, S100A1/A16 showed a significant correlation with the infiltration of CD8^+^ T cells, CD4^+^ T cells, macrophages, and dendritic cells. Interestingly, the expression of S100A12/B/Z was associated with the infiltration of these six immune cells (B cells, CD8 + T cells, CD4 + T cells, macrophages, neutrophil, and dendritic cells). Except for B cells, S100A8 and S100A9 was positively correlated with the infiltration of the other immune cells (CD8^+^ T cells, CD4^+^ T cells, macrophages, neutrophil, and dendritic cells). In conclusion, the above results suggested that the S100s may play an important role in the immune infiltration of PAAD.

### Differential Expression Analysis of Samples with High Expression of the Significant S100 Genes (top 25 percentile) vs Low Expression (bottom 25 percentile) Samples

**FIGURE 10:**
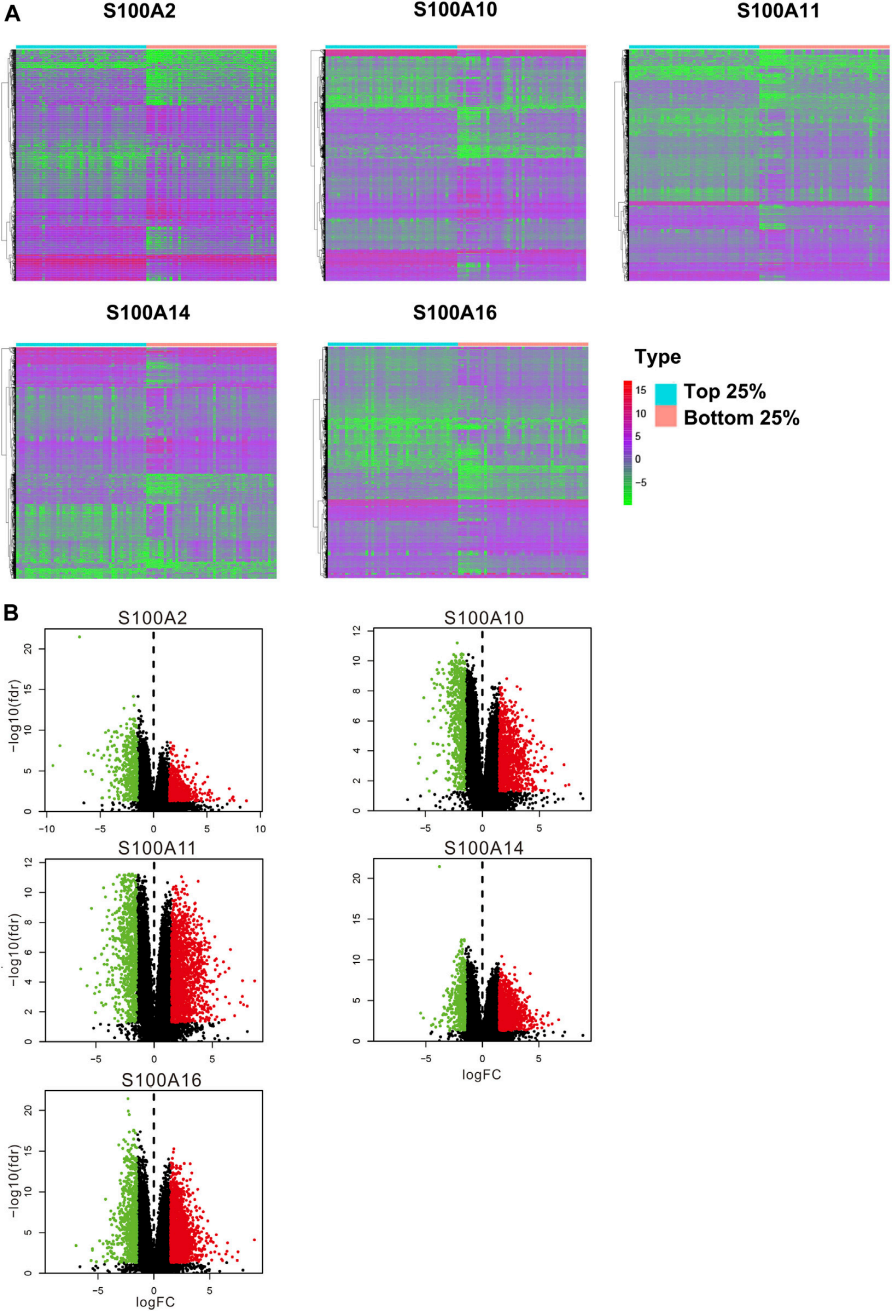
Differential expression analysis of samples with high expression of the significant S100 genes **(top 25%)** vs low expression **(bottom 25%)** samples (calculated data form TCGA-PAAD database, n=178). **(A) **Differentially expressed genes were displayed by heatmap. Wilcox test was used to analyze differentially expressed genes. The screening conditions are FDR=0.05 and logFC=1.5. The green on the heatmap indicates low expression and red indicates high expression. **(B)** Volcano plot. The green dot on the volcano plot represents down-regulated expression, and the red dot represents up-regulated expression.

**FIGURE 11:**
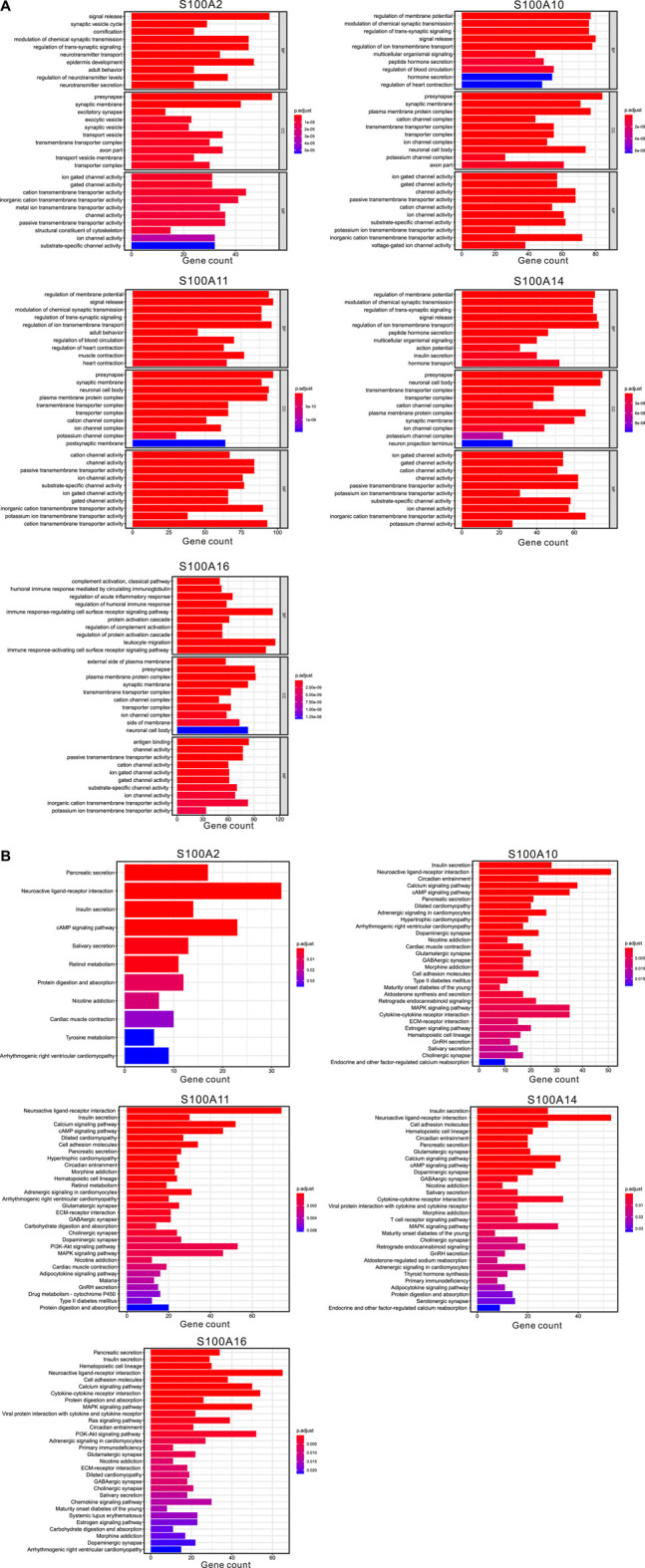
Functional enrichment analysis of differential genes in samples with significant S100 gene high expression **(top 25%)** and low expression **(bottom 25%)** samples (calculated data from TCGA-PAAD, n=178). **(A)** GO enrichment analysis (BP: biological process; CC: cellular component; MF: molecular function). **(B)** Top 30 most significant KEGG pathways.

**FIGURE 12:**
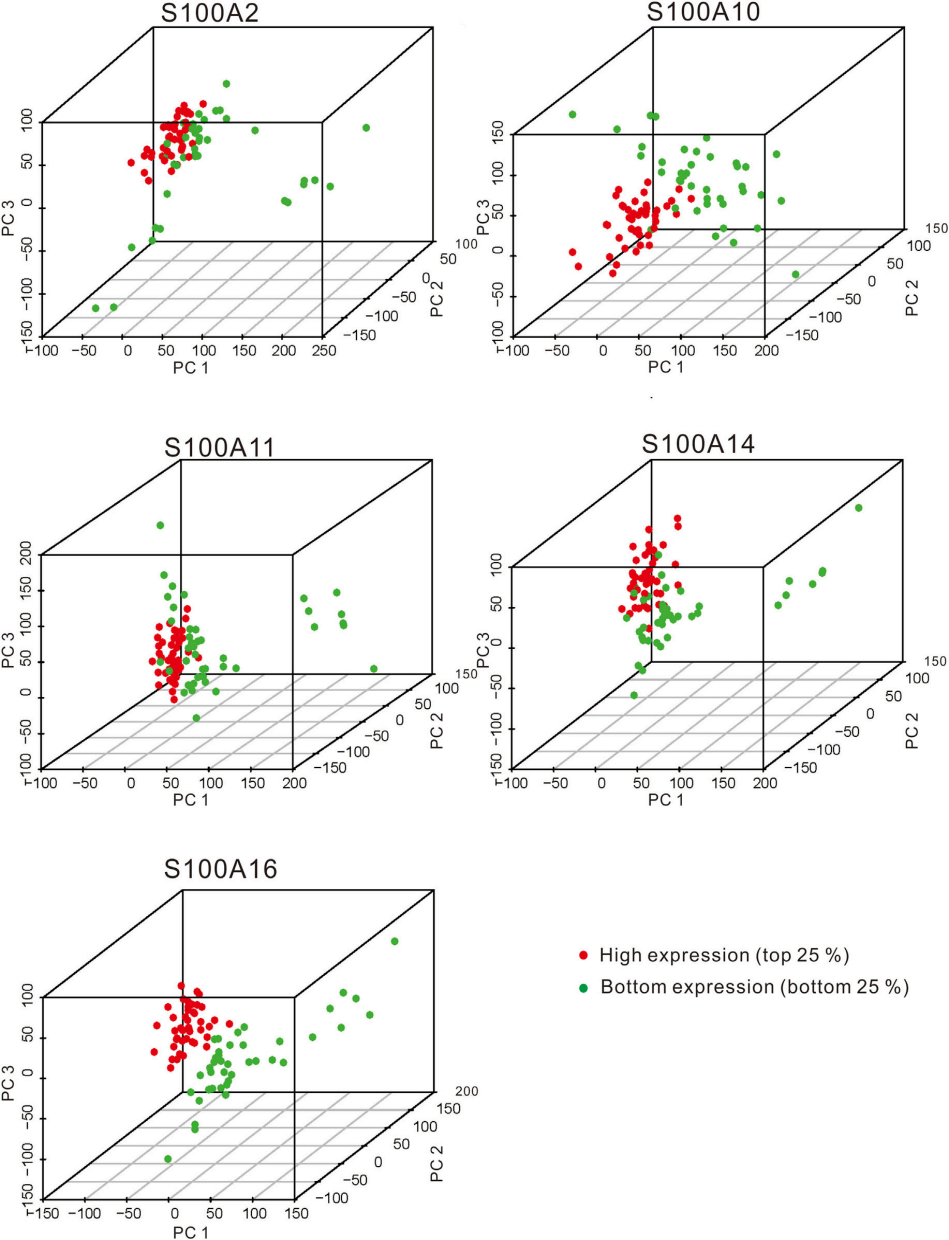
The PCA plots showed the genes in samples with significant S100 gene high expression **(top 25%)** and low expression **(bottom 25%)** samples (calculated data from TCGA-PAAD, n=178). The red dot and green dot indicate the smaples with the high expression of the significant S100 genes **(top 25 percentile)** and the low expression **(bottom 25 percentile)** samples, respectively.

We selected five S100 genes (S100A2/A10/A11/A14/A16) by using the above analysis, which were highly expressed in PAAD and associated with poor prognosis and chemoresistance of PAAD, for further differential expression analysis between the high (top 25 percentile) and low samples (bottom 25 percentile). We downloaded pancreatic cancer samples and normal samples from the TCGA and the GEXT database respectively. We used Wilcox. test to analyze differentially expressed genes in samples. The screening condition was FDR = 0.05 and logFC = 1.5. We found that the differentially expressed genes of these five S100 genes were 1,048, 1858, 2,492, 1,668, and 2,482, respectively (Figure 10A and Figure 10B). We enriched the GO and KEGG signal pathways of these differential genes respectively. We found that there were many similarities in the signal pathways enriched by these genes (Figure 11A and Figure 11B). We also found that there were significant differences between the high and low expression samples by using the principal component analysis (PCA). (Figure 12).

## Discussion

The transcription and protein expression levels of multiple S100s members have been confirmed to have changed in various of tumors (Allgöwer et al., 2020b). Emerging evidence displayed that the biological characteristics of most S100s members are complex and multifactorial, and these members are actively involved in the process of tumorigenesis and progression, such as tumor cell proliferation, angiogenesis, metastasis, immune escape and drug resistance (Donato, 2001; Chen et al., 2014; Bresnick et al., 2015; Moravkova et al., 2016; Zhang et al., 2017; Ma et al., 2019). In this study, we evaluated the expression, genomic mutation, prognostic value, potential function, relationship with immune infiltration, and involvement in the regulation of drug resistance of the S100s in PAAD. We hope that our results will help to reveal the role of the S100s in cancer and provide new theoretical knowledge for the identification of tumor prognostic biomarkers and therapeutic targets. We found that the transcription levels of 13 S100s members were overexpressed in PAAD patients. The high expression of S100A2/A3/A4/A5/A6/A10/A11/A13/A14/A16/P was positively associated with TP53 mutation. Combined with the transcriptional expression profile, we concluded that high mRNA expression of S100A2/A3/A10/A11/A14/A16 was correlated with poor OS, while high expression of S100B was related to better OS. Moreover, the high expression of S100A2/A6/A10/A11/A13/A14/A16 was resistant to small drugs. These findings suggested that S100A2/A10/A11/A14/A16 may have the potential to become a biomarker for prognosis or treatment of PAAD.

S100A2 is a well-studied S100s member in cancer, which is closely related to the occurrence of various human tumorigenesis (Wolf et al., 2011). S100A2 seems to have both carcinogenic and anticancer effects in cancer. Previous studies have shown that S100A2 plays an anticancer role in oral cancer, prostate cancer, breast cancer, and lung cancer, while it acts as a cancer promoter in ovarian cancer, gastric cancer, and esophageal squamous carcinoma (Liu et al., 2000; Rehman et al., 2005; Bulk et al., 2009; Donato et al., 2013; Gross et al., 2014; Bresnick et al., 2015). Overexpression of S100A2 was related to advanced histological grade, high T stage, and poor prognosis in pancreatic cancer (Zhuang et al., 2021). A large retrospective study suggests that S100A2 may have the potential to become a biomarker for predicting pancreatectomy or replication therapy in patients with pancreatic cancer (Jamieson et al., 2011; Bachet et al., 2013). S100A2 is regulated by cell cycle progression and tumor suppressor gene p53, and it can interact with RAGE *in vitro* (Leclerc et al., 2009; Donato et al., 2013). According to the signaling pathway activated by S100A2/RAGE in pancreatic cancer cells, S100A2 can be used as either a tumor suppressor or a tumor promoter (Leclerc et al., 2009). Our results showed that S100A2 was significantly up-regulated in PAAD. Moreover, high S100A2 mRNA expression was related to worse OS, drug resistance, and high TP53 mutation.

S100A10 plays an important role in the progression of various cancers. S100A10 is up-regulated by the Smad4-dependent transforming growth factor-β 1 signaling pathway (Bydoun et al., 2018a). Previous studies have shown that the overexpression of S100A10 is usually related to tumor size, pathological TNM stage, lymphovascular invasion, lymph node metastasis, poor prognosis, and drug resistance in many malignancies (Zhang et al., 2004; Saiki and Horii, 2019). Overexpression of S100A10 is associated with a poor prognosis of pancreatic ductal adenocarcinoma (Bydoun et al., 2018b). Meanwhile, Bydoun et al. (2018) demonstrated that S100A10 is involved in a new mechanism of plasminogen activation during the epithelial-mesenchymal transition (EMT) (Bydoun et al., 2018a). In our study, we found that the mRNA level of S100A10 was highly expressed in pancreatic cancer and was related to the high tumor stage. Overexpression of S100A10 is associated with poor prognosis, high TP53 mutation, and drug resistance. In addition, S100A10 may be related to the activation of RAS/MAPK, EMT, and SMAD4‐dependent TGF-β1 pathways.

S100A11 has been shown to play a dual role in tumors, such as inhibiting cancer of the bladder and kidney or promoting cancer of the pancreas (Yao et al., 2007; Donato et al., 2013; Gross et al., 2014; Bresnick et al., 2015). Previous studies have shown that S100A11 increases in the early stages of pancreatic cancer, but decreases as cancer progress (Ohuchida et al., 2006). Overexpression of S100A11 is associated with an unfavorable prognosis of patients with pancreatic ductal carcinoma undergoing surgical resection (Xiao et al., 2012). S100A11 can also promote the proliferation and viability of pancreatic cancer cells by up-regulating the PI3K/AKT signaling pathway, which is considered to be a promising new drug target for targeted therapy of pancreatic cancer (Xiao et al., 2018). In addition, S100A11 is considered as a molecular marker for early diagnosis of pancreatic cancer or for screening patients with high-risk lesions that have progressed to pancreatic cancer (Ohuchida et al., 2006). Our results displayed that S100A11 mRNA expression was significantly higher than that in normal pancreatic tissues. Overexpression of S100A11 is associated with tumor stage, drug resistance, high TP 53 mutation, and shorter overall survival.

S100A14 plays an important role in the occurrence and development of various human cancers (Zhao et al., 2013; Tanaka et al., 2015; Al-Ashkar and Zetoune, 2020; Li et al., 2020b; Zhu et al., 2021). Gene expression microarray showed that the S100A14 expression in pancreatic cancer tissues was significantly higher than that in corresponding non-tumor tissues (Ohuchida et al., 2006; Zhuang et al., 2021). Studies show that S100A14 protein is often overexpressed in pancreatic ductal adenocarcinoma (PDAC) cell lines and tissues. High expression of S100A14 was significantly correlated with advanced tumor stage and shorter overall survival in patients with pancreatic cancer (Ohuchida et al., 2006; Zhuang et al., 2021). Transient silencing of S100A14 can inhibit the proliferation, clone formation, migration, and invasion of high-level endogenous S100A14 cells (Ohuchida et al., 2006). Continuous knockout of S100A14 by transduction of lentivirus-carrying shRNAs inhibited the formation of subcutaneous tumors in nude mice and made PDAC cells more sensitive to gemcitabine treatment (Ohuchida et al., 2006). Our results indicate that S100A14 is highly expressed both in mRNA and protein levels of pancreatic cancer. High expression of S100A14 was associated with poor overall survival, tumor stage, high TP53 mutation, and drug resistance. In addition, S100A14 was negatively correlated with the infiltration of CD4 + T cells, macrophages, and dendritic cells.

S100A16 is a new member of the S100 protein family, which is functionally expressed in various tumors. The expression of S100A16 in PDAC was up-regulated, and it was negatively correlated with the prognosis of patients with PDAC (Zhuang et al., 2021). S100A16 can promote the proliferation, migration, and invasion of PDAC cells through AKT and ERK1/2 signaling pathways mediated by fibroblast growth factor 19 (FGF19) (Fang et al., 2021). S100A16 can also regulate the cell cycle and apoptosis of pancreatic cancer cells (Fang et al., 2021). Moreover, previous studies have shown that S100A16 can induce EMT and promote the metastasis of human PDAC cells by enhancing the expression of TWIST1 and activating the STAT3 signaling pathway (Li et al., 2021). Similar to these results, our results suggest that S100A16 is highly expressed in pancreatic cancer. High expression of S100A16 was associated with worse overall survival, tumor stage, high TP 53 mutation, and drug resistance.

In the current study, we also found that the expression of the S100s was correlated with the infiltration of certain immune cells in PAAD. Limited studies have been used to elucidate the role of the S100s in immune infiltration. Previous studies have shown that the higher expression of S1000A6/A10/A11/A14/A16 may damage the infiltration and cytotoxicity of CD8^+^ T cells by stimulating the adhesion-Ras signal transduction pathway in pancreatic cancer (Zhuang et al., 2021). S100A10 expressed on the surface of macrophages plays an important role in the interaction with tumor microenvironment and tumor growth (O'Connell et al., 2010). S100A16 is negatively correlated with immune activity (T cells, cytokines, chemokines, cell adhesion molecules, co-receptors, signal adapters, JAK/STAT pathway) and infiltration (macrophages and T cells), resulting in extensive immunosuppression (Chen et al., 2021). Consequently, our results contain an unconventional function of the S100s and provide new insights into the infiltration of immune cells in PAAD.

It is undeniable that our research has certain shortcomings. First, all the results are mined from the published database, so the quality of the database determines the reliability of our results. Second, our conclusions need to be verified by further experiments and multicenter clinical cohort samples. Third, this study did not explore the detailed mechanism of the predicted S100s members in pancreatic cancer, especially the impact on tumor immune microenvironment and drug sensitivity. To solve these problems, further *in vivo* and *in vitro* experiments are needed to verify these results.

## Conclusion

In this study, we systemically investigated the transcription and protein level, genetic mutations, prognostic value, enriched signal pathways, and the correlation with immune infiltration cells of each S100s member in PAAD patients. We evaluated the expression, genomic mutation, prognostic value, potential function, relationship with immune infiltration, and involvement in the regulation of drug resistance of the S100s in PAAD. We found that 13 S100s members were overexpressed in PAAD patients. Combined with the transcriptional expression profile, we concluded that high mRNA expression of S100A2/A3/A10/A11/A14/A16 were significantly correlated with the poor OS of PAAD, while a high expression of S100B is favorable to the prognosis of PAAD. Overexpression of S100A2/A3/A4/A5/A6/A10/A11/A13/A14/A16/P in pancreatic cancer is positively correlated with TP53 mutation, while the high expression of S100A1/A12/B/Z is negatively correlated with TP53 mutation. Immuno-infiltration analysis showed that the mRNA levels of S100A10/A11/A14/A16 were significantly correlated with the infiltration degree of macrophages in PAAD. Moreover, PAAD patients expressing high levels of S100A2/A6/A10/A11/A13/A14/A16 were resistant to small molecule drugs. These findings suggested that S100A2/A10/A11/A14/A16 may be prognostic and therapeutic targets of PAAD. We hope that our results will help to reveal the role of the S100s in cancers and provide new theoretical knowledge for identifying tumor prognostic biomarkers and therapeutic targets.

## Data Availability

The datasets presented in this study can be found in online repositories. The names of the repository/repositories and accession number(s) can be found in the article/Supplementary Material.

## Abbreviations

AKT, protein kinase B; AR, androgen receptor; CPTAC, clinical proteomic tumor analysis consortium; CTRP, clinical trials reporting program; DEG, differentially expressed gene; EMT, epithelial-mesenchymal transition; ER, estrogen receptor; GDSC, genomics of drug sensitivity in cancer; GEPIA, gene expression profiling interactive; GO, gene ontology; GTEx, genotype-Tissue expression; HPA, the human protein atlas; IHC, Immunohistochemistry; KEGG, kyoto encyclopedia of genes and genomes; MAPK, mitogen-activated protein kinase; mTOR, mechanistic target of rapamycin; OS, overall survival; PAAD, pancreatic adenocarcinoma; PCA, principal component analysis; PI3K, phosphoinositide 3-kinase; RTK, receptor tyrosine kinase; S100s, S100 family; TCGA, the cancer genome atlas; TPM, transcripts per million; TSC, Tuberous sclerosis complex.

## References

[B1] Adamska A. Elaskalani O. Emmanouilidi A. Kim M. Abdol Razak N. B. Metharom P. (2018). Molecular and Cellular Mechanisms of Chemoresistance in Pancreatic Cancer. Adv. Biol. Regul. 68, 77–87. 10.1016/j.jbior.2017.11.007 29221990

[B2] Al-Ashkar N. Zetoune A. B. (2020). S100A14 Serum Level and its Correlation with Prognostic Factors in Breast Cancer. J. Egypt. Natl. Canc Inst. 32 (1), 37. 10.1186/s43046-020-00048-y 32984913 PMC13317075

[B3] Allgöwer C. Kretz A. L. von Karstedt S. Wittau M. Henne-Bruns D. Lemke J. (2020). Friend or Foe: S100 Proteins in Cancer. Cancers 12 (8), 2037. 10.3390/cancers12082037 32722137 PMC7465620

[B4] Allgöwer C. Kretz A.-L. von Karstedt S. Wittau M. Henne-Bruns D. Lemke J. (2020). Friend or Foe: S100 Proteins in Cancer. Cancers 12 (8), 2037. 10.3390/cancers12082037 32722137 PMC7465620

[B5] Bachet J. B. Maréchal R. Demetter P. Bonnetain F. Cros J. Svrcek M. (2013). S100A2 Is a Predictive Biomarker of Adjuvant Therapy Benefit in Pancreatic Adenocarcinoma. Eur. J. Cancer 49 (12), 2643–2653. 10.1016/j.ejca.2013.04.017 23726265

[B6] Bresnick A. R. Weber D. J. Zimmer D. B. (2015). S100 Proteins in Cancer. Nat. Rev. Cancer 15 (2), 96–109. 10.1038/nrc3893 25614008 PMC4369764

[B7] Bulk E. Sargin B. Krug U. Hascher A. Jun Y. Knop M. (2009). S100A2 Induces Metastasis in Non-small Cell Lung Cancer. Clin. Cancer Res. 15 (1), 22–29. 10.1158/1078-0432.CCR-08-0953 19118029

[B8] Bydoun M. Sterea A. Liptay H. Uzans A. Huang W. Y. Rodrigues G. J. (2018). S100A10, a Novel Biomarker in Pancreatic Ductal Adenocarcinoma. Mol. Oncol. 12 (11), 1895–1916. 10.1002/1878-0261.12356 30009399 PMC6210040

[B9] Bydoun M. Sterea A. Weaver I. C. G. Bharadwaj A. G. Waisman D. M. (2018). A Novel Mechanism of Plasminogen Activation in Epithelial and Mesenchymal Cells. Sci. Rep. 8 (1), 14091. 10.1038/s41598-018-32433-y 30237490 PMC6148250

[B10] Camara R. Ogbeni D. Gerstmann L. Ostovar M. Hurer E. Scott M. (2020). Discovery of Novel Small Molecule Inhibitors of S100P with *In Vitro* Anti-metastatic Effects on Pancreatic Cancer Cells. Eur. J. Med. Chem. 203, 112621. 10.1016/j.ejmech.2020.112621 32707527 PMC7501730

[B11] Chen H. Xu C. Jin Q. Liu Z. (2014). S100 Protein Family in Human Cancer. Am. J. Cancer Res. 4 (2), 89–115. 24660101 PMC3960449

[B12] Chen T. Xia D. M. Qian C. Liu S. R. (2021). Integrated Analysis Identifies S100A16 as a Potential Prognostic Marker for Pancreatic Cancer. Am. J. Transl Res. 13 (5), 5720–5730. 34150181 PMC8205789

[B13] de Visser K. E. Eichten A. Coussens L. M. (2006). Paradoxical Roles of the Immune System during Cancer Development. Nat. Rev. Cancer 6 (1), 24–37. 10.1038/nrc1782 16397525

[B14] Donato R. (1999). Functional Roles of S100 Proteins, Calcium-Binding Proteins of the EF-Hand Type. Biochim. Biophys. Acta (Bba) - Mol. Cel Res. 1450 (3), 191–231. 10.1016/S0167-4889(99)00058-0 10395934

[B15] Donato R. R. Cannon B. Sorci G. Riuzzi F. Hsu K. Weber D. J. (2013). Functions of S100 Proteins. Curr. Mol. Med. 13 (1), 24–57. 10.2174/156652413804486214 22834835 PMC3707951

[B16] Donato R. (2001). S100: a Multigenic Family of Calcium-Modulated Proteins of the EF-Hand Type with Intracellular and Extracellular Functional Roles. Int. J. Biochem. Cel Biol. 33 (7), 637–668. 10.1016/s1357-2725(01)00046-2 11390274

[B17] Engelkamp D. Schafer B. W. Mattei M. G. Erne P. Heizmann C. W. (1993). Six S100 Genes Are Clustered on Human Chromosome 1q21: Identification of Two Genes Coding for the Two Previously Unreported Calcium-Binding Proteins S100D and S100E. Proc. Natl. Acad. Sci. 90 (14), 6547–6551. 10.1073/pnas.90.14.6547 8341667 PMC46969

[B18] Fang D. Zhang C. Xu P. Liu Y. Mo X. Sun Q. (2021). S100A16 Promotes Metastasis and Progression of Pancreatic Cancer through FGF19-Mediated AKT and ERK1/2 Pathways. Cell Biol Toxicol 37, 555–571. 10.1007/s10565-020-09574-w 33389337

[B19] Gao J. Aksoy B. A. Dogrusoz U. Dresdner G. Gross B. Sumer S. O. (2013). Integrative Analysis of Complex Cancer Genomics and Clinical Profiles Using the cBioPortal. Sci. Signal. 6 (269). 10.1126/scisignal.2004088 PMC4160307 23550210

[B20] Goh J. Y. Feng M. Wang W. Oguz G. Yatim S. M. J. M. Lee P. L. (2017). Chromosome 1q21.3 Amplification Is a Trackable Biomarker and Actionable Target for Breast Cancer Recurrence. Nat. Med. 23 (11), 1319–1330. 10.1038/nm.4405 28967919

[B21] Gross S. R. Sin C. G. T. Barraclough R. Rudland P. S. (2014). Joining S100 Proteins and Migration: for Better or for Worse, in Sickness and in Health. Cell. Mol. Life Sci. 71 (9), 1551–1579. 10.1007/s00018-013-1400-7 23811936 PMC11113901

[B22] Ilic M. Ilic I. (2016). Epidemiology of Pancreatic Cancer. Wjg 22 (44), 9694–9705. 10.3748/wjg.v22.i44.9694 27956793 PMC5124974

[B23] Jamieson N. B. Denley S. M. Logue J. MacKenzie D. J. Foulis A. K. Dickson E. J. (2011). A Prospective Comparison of the Prognostic Value of Tumor- and Patient-Related Factors in Patients Undergoing Potentially Curative Surgery for Pancreatic Ductal Adenocarcinoma. Ann. Surg. Oncol. 18 (8), 2318–2328. 10.1245/s10434-011-1560-3 21267785

[B24] Kamisawa T. Wood L. D. Itoi T. Takaori K. (2016). Pancreatic Cancer. The Lancet 388 (10039), 73–85. 10.1016/S0140-6736(16)00141-0 26830752

[B25] Lai E. Puzzoni M. Ziranu P. Pretta A. Impera V. Mariani S. (2019). New Therapeutic Targets in Pancreatic Cancer. Cancer Treat. Rev. 81, 101926. Artn 101926. 10.1016/J.Ctrv.2019.101926 31739115

[B26] Leclerc E. Fritz G. Vetter S. W. Heizmann C. W. (2009). Binding of S100 Proteins to RAGE: an Update. Biochim. Biophys. Acta (Bba) - Mol. Cel Res. 1793 (6), 993–1007. 10.1016/j.bbamcr.2008.11.016 19121341

[B27] Lei X. Lei Y. Li J. K. Du W. X. Li R. G. Yang J. (2020). Immune Cells within the Tumor Microenvironment: Biological Functions and Roles in Cancer Immunotherapy. Cancer Lett. 470, 126–133. 10.1016/j.canlet.2019.11.009 31730903

[B28] Li T. Fu J. Zeng Z. Cohen D. Li J. Chen Q. (2020). TIMER2.0 for Analysis of Tumor-Infiltrating Immune Cells. Nucleic Acids Res. 48 (W1), W509–W514. 10.1093/nar/gkaa407 32442275 PMC7319575

[B29] Li T. Ren T. Huang C. Li Y. Yang P. Che G. (2021). S100A16 Induces Epithelial-Mesenchymal Transition in Human PDAC Cells and Is a New Therapeutic Target for Pancreatic Cancer Treatment that Synergizes with Gemcitabine. Biochem. Pharmacol. 189, 114396. 10.1016/j.bcp.2020.114396 33359364

[B30] Li X. Wang M. Gong T. Lei X. Hu T. Tian M. (2020). A S100A14-Ccl2/cxcl5 Signaling axis Drives Breast Cancer Metastasis. Theranostics 10 (13), 5687–5703. 10.7150/thno.42087 32483412 PMC7255008

[B31] Liu C. J. Hu F. F. Xia M. X. Han L. Zhang Q. Guo A. Y. (2018). GSCALite: a Web Server for Gene Set Cancer Analysis. Bioinformatics 34 (21), 3771–3772. 10.1093/bioinformatics/bty411 29790900

[B32] Liu D. Rudland P. S. Sibson D. R. Platt-Higgins A. Barraclough R. (2000). Expression of Calcium-Binding Protein S100A2 in Breast Lesions. Br. J. Cancer 83 (11), 1473–1479. 10.1054/bjoc.2000.1488 11076656 PMC2363420

[B33] Ma N. Zhu L. Yang L. Cui Y. Zhan Y. (2019). Prognostic Values of S100 Family mRNA Expression in Ovarian Cancer. Cbm 25 (1), 67–78. 10.3233/CBM-182276 PMC13082422 31033462

[B34] Marchesi F. Piemonti L. Mantovani A. Allavena P. (2010). Molecular Mechanisms of Perineural Invasion, a Forgotten Pathway of Dissemination and Metastasis. Cytokine Growth Factor. Rev. 21 (1), 77–82. 10.1016/j.cytogfr.2009.11.001 20060768

[B35] Marenholz I. Heizmann C. W. Fritz G. (2004). S100 Proteins in Mouse and Man: from Evolution to Function and Pathology (Including an Update of the Nomenclature). Biochem. Biophysical Res. Commun. 322 (4), 1111–1122. 10.1016/j.bbrc.2004.07.096 15336958

[B36] Marenholz I. Lovering R. C. Heizmann C. W. (2006). An Update of the S100 Nomenclature. Biochim. Biophys. Acta (Bba) - Mol. Cel Res. 1763 (11), 1282–1283. 10.1016/j.bbamcr.2006.07.013 16938360

[B37] Mishra N. K. Southekal S. Guda C. (2019). Survival Analysis of Multi-Omics Data Identifies Potential Prognostic Markers of Pancreatic Ductal Adenocarcinoma. Front. Genet. 10, 10. 10.3389/fgene.2019.00624 31379917 PMC6659773

[B38] Moravkova P. Kohoutova D. Rejchrt S. Cyrany J. Bures J. (20162016). Role of S100 Proteins in Colorectal Carcinogenesis. Gastroenterol. Res. Pract. 2016, 1–7. 10.1155/2016/2632703 PMC4736765 26880885

[B39] O'Connell P. A. Surette A. P. Liwski R. S. Svenningsson P. Waisman D. M. (2010). S100A10 Regulates Plasminogen-dependent Macrophage Invasion. Blood 116 (7), 1136–1146. 10.1182/blood-2010-01-264754 20424186

[B40] Ohuchida K. Mizumoto K. Ohhashi S. Yamaguchi H. Konomi H. Nagai E. (2006). S100A11, a Putative Tumor Suppressor Gene, Is Overexpressed in Pancreatic Carcinogenesis. Clin. Cancer Res. 12 (18), 5417–5422. 10.1158/1078-0432.CCR-06-0222 17000675

[B41] Rehman I. Cross S. S. Catto J. W. F. Leiblich A. Mukherjee A. Azzouzi A.-R. (2005). Promoter Hyper-Methylation of Calcium Binding Proteins S100A6 and S100A2 in Human Prostate Cancer. Prostate 65 (4), 322–330. 10.1002/pros.20302 16015609

[B42] Saiki Y. Horii A. (2019). Multiple Functions of S100A10, an Important Cancer Promoter. Pathol. Int. 69 (11), 629–636. 10.1111/pin.12861 31612598

[B43] Salama I. Malone P. S. Mihaimeed F. Jones J. L. (2008). A Review of the S100 Proteins in Cancer. Eur. J. Surg. Oncol. (Ejso) 34 (4), 357–364. 10.1016/j.ejso.2007.04.009 17566693

[B44] Schäfer B. W. Wicki R. Engelkamp D. Mattei M.-g. Heizmann C. W. (1995). Isolation of a YAC Clone Covering a Cluster of Nine S100 Genes on Human Chromosome 1q21: Rationale for a New Nomenclature of the S100 Calcium-Binding Protein Family. Genomics 25 (3), 638–643. 10.1016/0888-7543(95)80005-7 7759097

[B45] Sighoko D. Curado M. P. Bourgeois D. Mendy M. Hainaut P. Bah E. (2011). Increase in Female Liver Cancer in the Gambia, West Africa: Evidence from 19 Years of Population-Based Cancer Registration (1988-2006). PLoS One 6 (4), e18415. 10.1371/journal.pone.0018415 21490972 PMC3072390

[B46] Sung H. Ferlay J. Siegel R. L. Laversanne M. Soerjomataram I. Jemal A. (2021). Global Cancer Statistics 2020: GLOBOCAN Estimates of Incidence and Mortality Worldwide for 36 Cancers in 185 Countries. CA A. Cancer J. Clin. 71 (3), 209–249. 10.3322/caac.21660 33538338

[B47] Szklarczyk D. Gable A. L. Lyon D. Junge A. Wyder S. Huerta-Cepas J. (2019). STRING V11: Protein-Protein Association Networks with Increased Coverage, Supporting Functional Discovery in Genome-wide Experimental Datasets. Nucleic Acids Res. 47 (D1), D607–d613. 10.1093/nar/gky1131 30476243 PMC6323986

[B48] Tanaka M. Ichikawa-Tomikawa N. Shishito N. Nishiura K. Miura T. Hozumi A. (2015). Co-expression of S100A14 and S100A16 Correlates with a Poor Prognosis in Human Breast Cancer and Promotes Cancer Cell Invasion. BMC Cancer 15, 53. 10.1186/s12885-015-1059-6 25884418 PMC4348405

[B49] Tang Z. Li C. Kang B. Gao G. Li C. Zhang Z. (2017). GEPIA: a Web Server for Cancer and normal Gene Expression Profiling and Interactive Analyses. Nucleic Acids Res. 45 (W1), W98–W102. 10.1093/nar/gkx247 28407145 PMC5570223

[B50] Thul P. J. Lindskog C. (2018). The Human Protein Atlas: A Spatial Map of the Human Proteome. Protein Sci. 27 (1), 233–244. 10.1002/pro.3307 28940711 PMC5734309

[B51] Warde-Farley D. Donaldson S. L. Comes O. Zuberi K. Badrawi R. Chao P. (2010). The GeneMANIA Prediction Server: Biological Network Integration for Gene Prioritization and Predicting Gene Function. Nucleic Acids Res. 38 (Web Server issue), W214–W220. 10.1093/nar/gkq537 20576703 PMC2896186

[B52] Wolf S. Haase-Kohn C. Pietzsch J. (2011). S100A2 in Cancerogenesis: a Friend or a Foe? Amino Acids 41 (4), 849–861. 10.1007/s00726-010-0623-2 20521072

[B53] Xiao M.-B. Jiang F. Ni W.-K. Chen B.-Y. Lu C.-H. Li X.-Y. (2012). High Expression of S100A11 in Pancreatic Adenocarcinoma Is an Unfavorable Prognostic Marker. Med. Oncol. 29 (3), 1886–1891. 10.1007/s12032-011-0058-y 21912994

[B54] Xiao M. Li T. Ji Y. Jiang F. Ni W. Zhu J. (2018). S100A11 Promotes Human Pancreatic Cancer PANC-1 Cell Proliferation and Is Involved in the PI3K/AKT Signaling Pathway. Oncol. Lett. 15 (1), 175–182. 10.3892/ol.2017.7295 29375710 PMC5766079

[B55] Xue T. C. Zhang B. H. Ye S. L. Ren Z. G. (2015). Differentially Expressed Gene Profiles of Intrahepatic Cholangiocarcinoma, Hepatocellular Carcinoma, and Combined Hepatocellular-Cholangiocarcinoma by Integrated Microarray Analysis. Tumor Biol. 36 (8), 5891–5899. 10.1007/s13277-015-3261-1 25712376

[B56] Yao R. Davidson D. D. Lopez-Beltran A. MacLennan G. T. Montironi R. Cheng L. (2007). The S100 Proteins for Screening and Prognostic Grading of Bladder Cancer. Histol. Histopathol 22 (9), 1025–1032. 10.14670/HH-22.1025 17523080

[B57] Zhang C. Zou Y. Zhu Y. Liu Y. Feng H. Niu F. (2021). Three Immune-Related Prognostic mRNAs as Therapeutic Targets for Pancreatic Cancer. Front. Med. 8. Artn 649326. 10.3389/Fmed.2021.649326 PMC8047149 33869254

[B58] Zhang L. Fogg D. K. Waisman D. M. (2004). RNA Interference-Mediated Silencing of the S100A10 Gene Attenuates Plasmin Generation and Invasiveness of Colo 222 Colorectal Cancer Cells. J. Biol. Chem. 279 (3), 2053–2062. 10.1074/jbc.M310357200 14570893

[B59] Zhang S. Wang Z. Liu W. Lei R. Shan J. Li L. (2017). Distinct Prognostic Values of S100 mRNA Expression in Breast Cancer. Sci. Rep. 7, 39786. 10.1038/srep39786 28051137 PMC5209742

[B60] Zhao F.-T. Jia Z.-S. Yang Q. Song L. Jiang X. J. (2013). S100A14 Promotes the Growth and Metastasis of Hepatocellular Carcinoma. Asian Pac. J. Cancer Prev. 14 (6), 3831–3836. 10.7314/apjcp.2013.14.6.3831 23886191

[B61] Zhu H. Gao W. Li X. Yu L. Luo D. Liu Y. (2021). S100A14 Promotes Progression and Gemcitabine Resistance in Pancreatic Cancer. Pancreatology 21 (3), 589–598. 10.1016/j.pan.2021.01.011 33579599

[B62] Zhuang H. Chen X. Dong F. Zhang Z. Zhou Z. Ma Z. (2021). Prognostic Values and Immune Suppression of the S100A Family in Pancreatic Cancer. J. Cel Mol Med 25 (6), 3006–3018. 10.1111/jcmm.16343 PMC7957204 33580614

