# Corrigendum: Prognostic Values and Clinical Significance of S100 Family Member’s Individualized mRNA Expression in Pancreatic Adenocarcinoma

**DOI:** 10.3389/fgene.2021.838600

**Published:** 2022-01-25

**Authors:** Xiaomin Li, Ning Qiu, Qijuan Li

**Affiliations:** ^1^ Guangzhou Women and Children’s Medical Center, Guangzhou Medical University, Guangzhou, China; ^2^ Key Laboratory of Ocean and Marginal Sea Geology, Guangdong Southern Marine Science and Engineering Laboratory (Guangzhou), South China Sea Institute of Oceanology, Innovation Academy of South China Sea Ecology and Environmental Engineering, Chinese Academy of Sciences, Guangzhou, China; ^3^ Department of Clinical Laboratory, The Fifth Affiliated Hospital of Sun Yat-sen University, Zhuhai, China

**Keywords:** mRNA expression, S100 family, pancreatic cancer, biomarker, prognosis

There was an error in the **Funding** statement. The correct numbers for the Key Special Project for Introduced Talents Team of Southern Marine Science and Engineering Guangdong Laboratory are GML2019ZD0104 and 2019BT02H594, for Foundation of Guangzhou Women and Children’s Medical Center is 2020BS006, and the Natural Science Foundation of Guangdong Province is 2021A1515011526.

In the original article, there was a mistake in the legend for **Figure 1** as published. The text in the last sentence of the Figure Legend “S100A2/A4/A6/A10/A11A13/14/A16/P” is missing a slash. The correct text is “S100A2/A4/A6/A10/A11/A13/14/A16/P.”

In the original article, there was a mistake in Figure 4 as published. The subplot of the S100A9, S100A10, and S100A11 in Figure 4 was duplicated. The corrected Figure 4 appears below.

**FIGURE 4:**
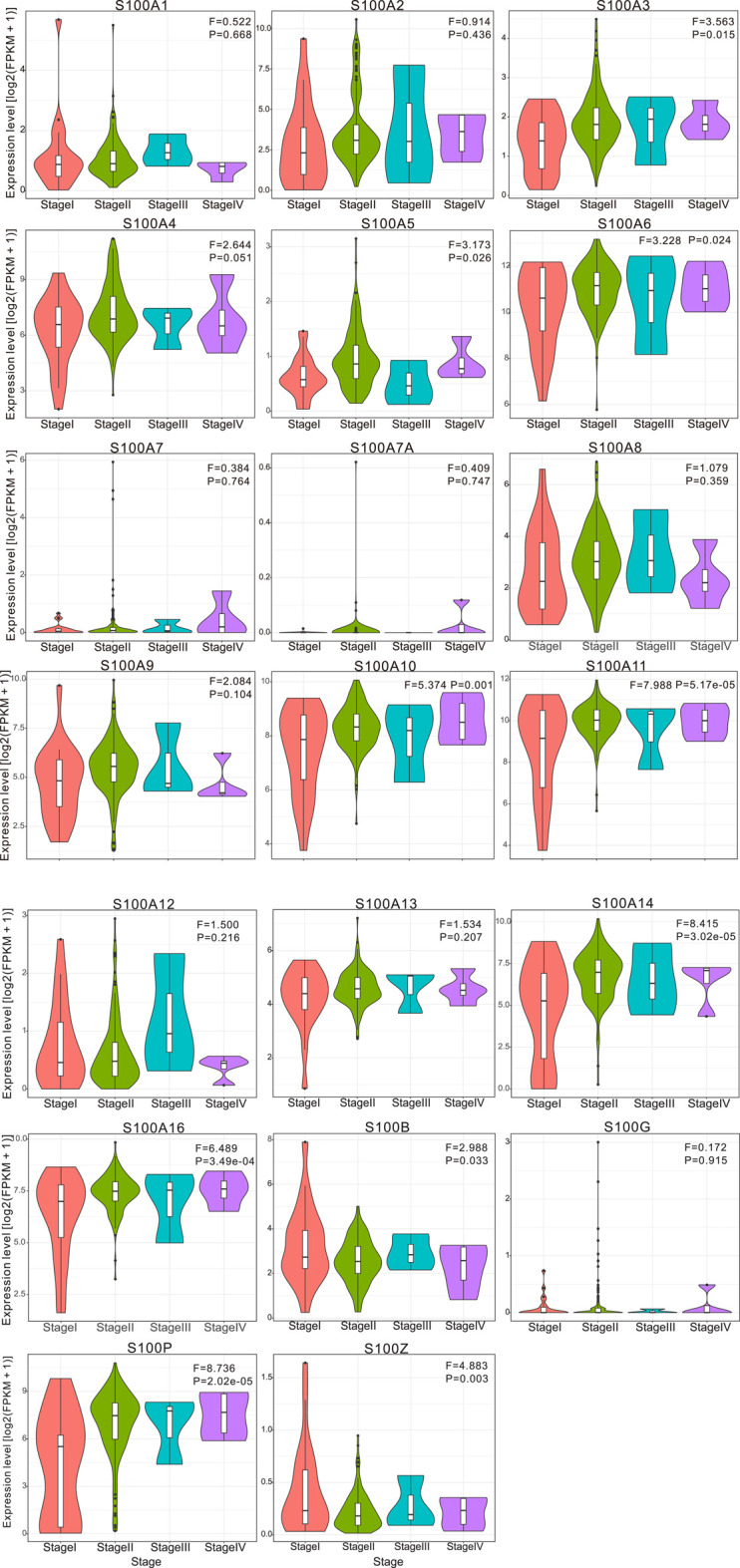
Violin plot demonstrated the correlation between S100s transcription level and tumor stages in patients with PAAD (calculated data form TCGAPAAD). The difference of individual S100s gene expression in each stage was analyzed by one-way ANOVA, in which Pr (>F) < 0.05 was considered to be statistically significant. The number of pancreatic cancer samples at each tumor stage was 21 cases in stage I, 146 in stage II, 3 in stage III, and 4 in stage IV. Abbreviation: F value, the statistical value of F test; Pr (>F), *p*-value

In the original article, there was a mistake in Figure 5 as published. The font size for the y-axis coordinate of S100B was inconsistent with the others, and the y-axis label for S100A12 was redundant. The corrected Figure 5 appears below.

**FIGURE 5:**
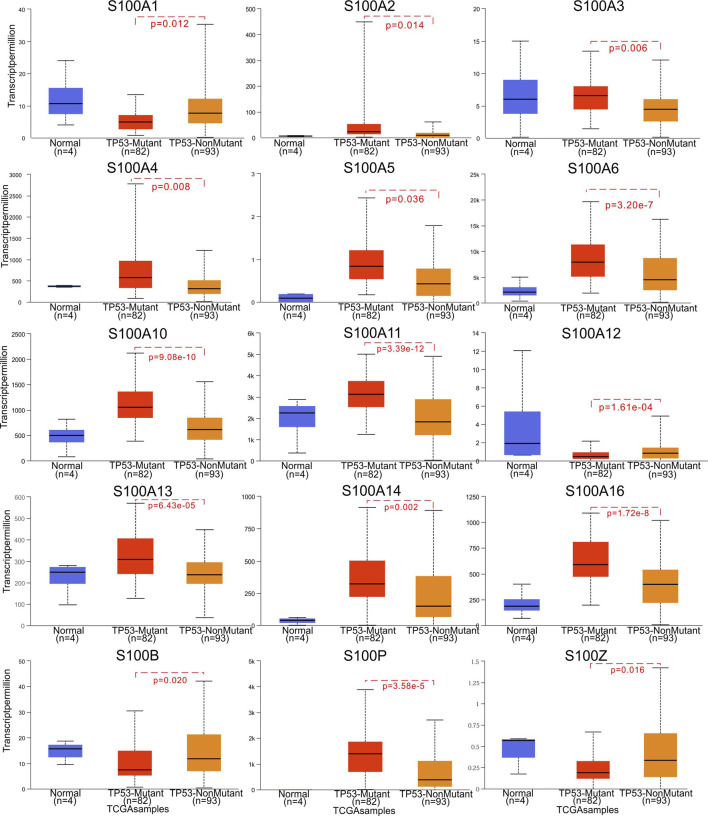
Correlation between S100s mRNA expression and TP53 mutation in PAAD (UALCAN). The significance of difference between TP53-mutant and TP53- nonmutant estimated by Student’s t-test with p value. The sample number N and the Student’s t-test p value are marked on the figure, respectively.

In the original article, there was a mistake in Figure 8 as published. It was missing part labels, and the legend erroneously included a part (C). The corrected Figure 8 appears below.

**FIGURE 8:**
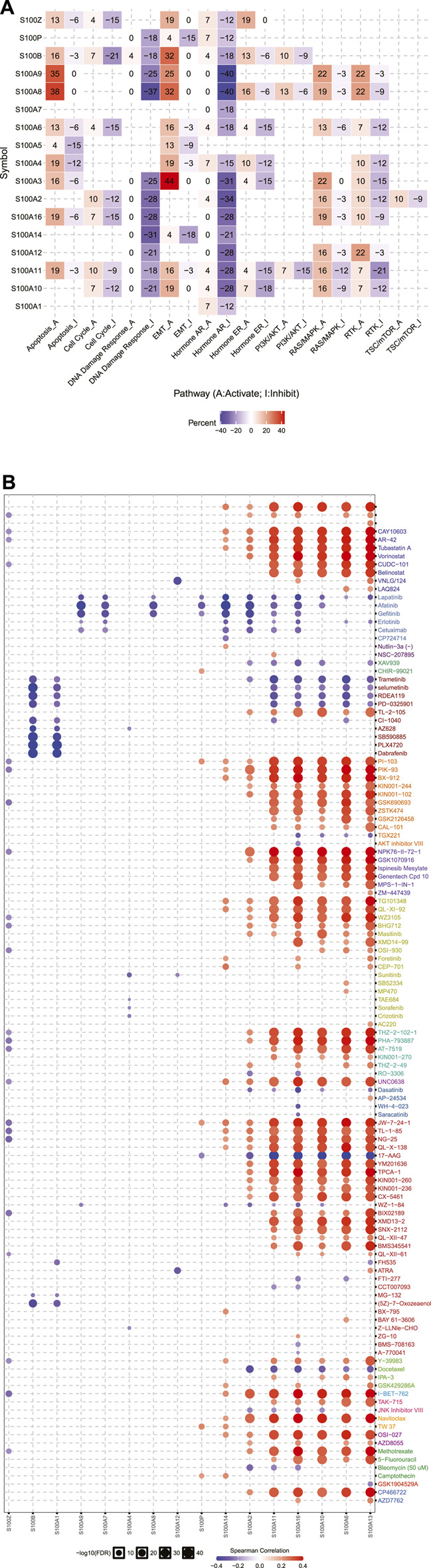
Cancer pathway activity and drug sensitivity analysis of the S100s in PAAD (GSCALite, n=178). **(A)** The role of the S100s in the famous cancer-related pathways. **(B)** Spearman correlation showed that S100 gene expression was related to small drugs. Negative correlation means that high gene expression is sensitive to the drug, and vice versa.

In the original article, there was a mistake in Figure 10A as published. The top label of the fourth subgraph of Figure 10A, “S100A10,” was incorrectly marked. The correct label: “S100A14.” In the original article, there was a mistake in Figure 10B as published. The top label of the fourth subgraph of Figure 10B, “S100A10,” was incorrectly marked. The correct label: “S100A14.” The corrected Figure 10A,B appears below.

**FIGURE 10:**
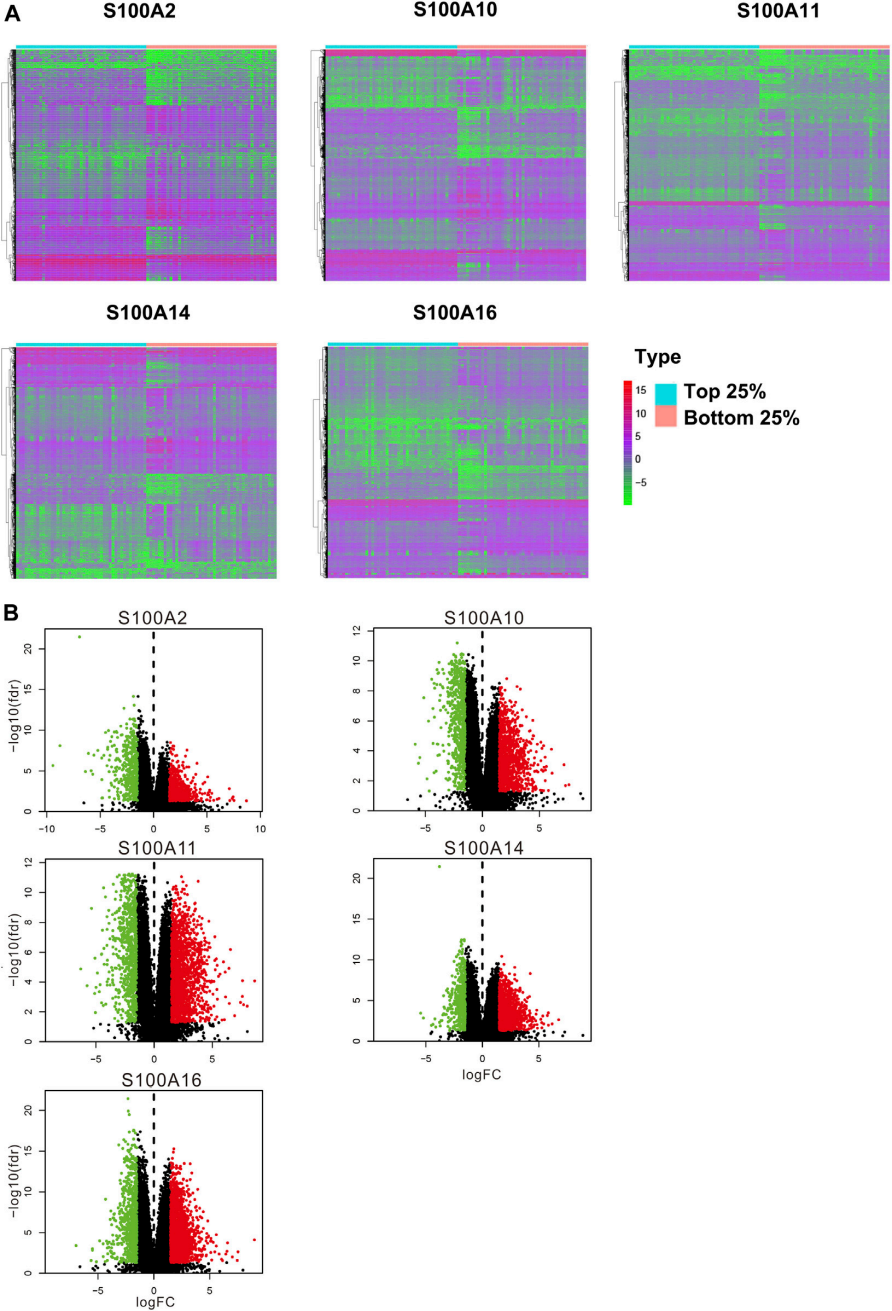
Differential expression analysis of samples with high expression of the significant S100 genes **(top 25%)** vs low expression **(bottom 25%)** samples (calculated data form TCGA-PAAD database, n = 178). **(A)** Differentially expressed genes were displayed by heatmap. Wilcox test was used to analyze differentially expressed genes. The screening conditions are FDR = 0.05 and logFC = 1.5. The green on the heatmap indicates low expression and red indicates high expression. **(B)** Volcano plot. The green dot on the volcano plot represents down-regulated expression, and the red dot represents up-regulated expression.

In the original article, there was a mistake in Figure 12 as published. The top label of the right subgraph in the Line 2, “S100A10,” was incorrectly marked. The correct label: “S100A14.” The corrected Figure 12 appears below.

**FIGURE 12:**
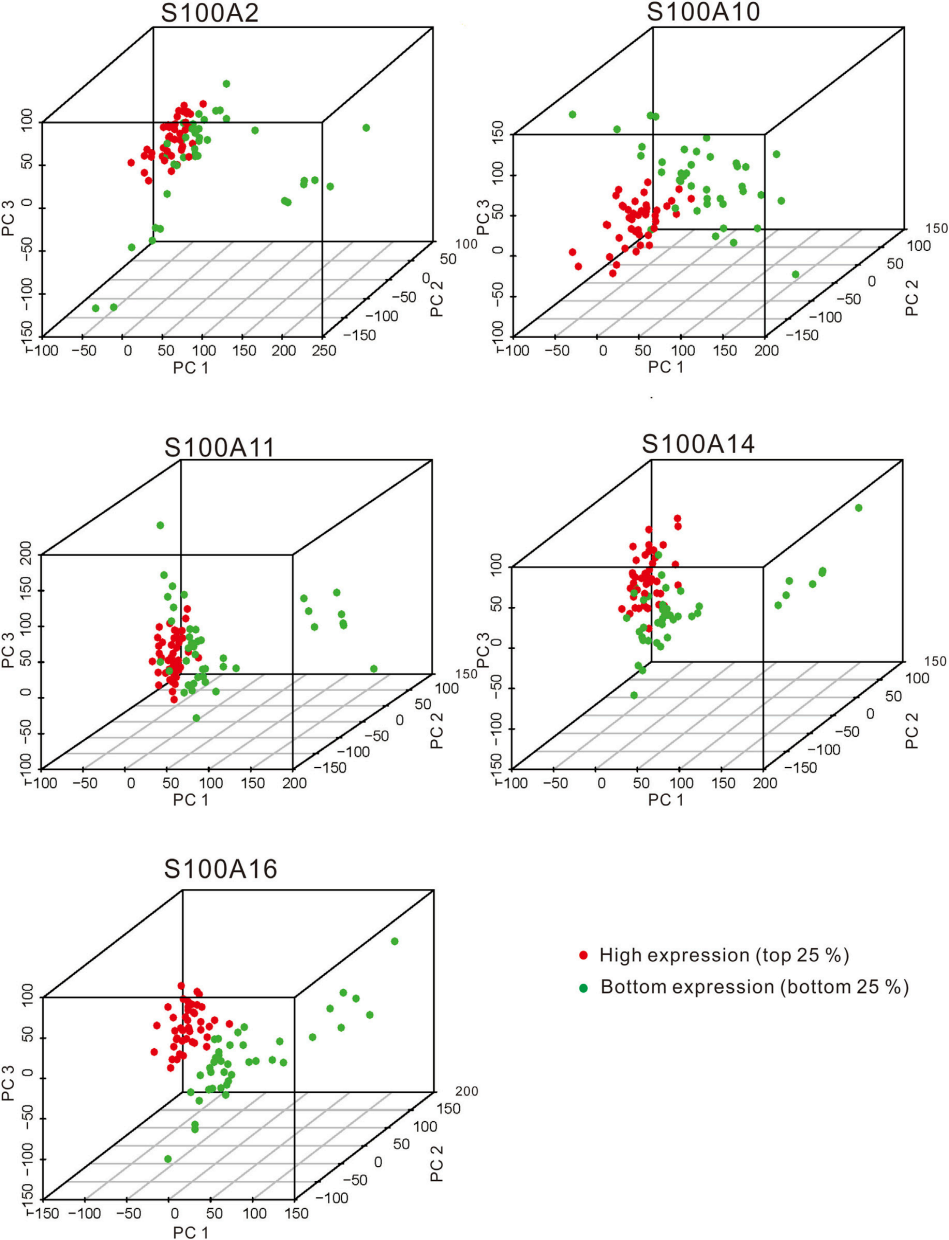
The PCA plots showed the genes in samples with significant S100 gene high expression **(top 25%)** and low expression **(bottom 25%)** samples (calculated data from TCGA-PAAD, n = 178). The red dot and green dot indicate the smaples with the high expression of the significant S100 genes **(top 25 percentile)** and the low expression **(bottom 25 percentile)** samples, respectively.

A correction has been made to **Results, Differential Expression Analysis of Samples with High Expression of the Significant S100 Genes (top 25 percentile) vs Low Expression (bottom 25 percentile) Samples**, paragraph 1, first sentence. The corrected sentence appears below:

We selected five S100 genes (S100A2/A10/A11/A14/A16) by using the above analysis, which were highly expressed in PAAD and associated with poor prognosis and chemoresistance of PAAD, for further differential expression analysis between the high (top 25 percentile) and low samples (bottom 25 percentile).

A correction has been made to **Discussion**, paragraph 1, sentence 7. The corrected sentence appears below:

The high expression of S100A2/A3/A4/A5/A6/A10/A11/A13/A14/A16/P was positively associated with TP53 mutation.

A correction has been made to **Conclusion**, paragraph 1, sentence 5. The corrected sentence appears below:

Overexpression of S100A2/A3/A4/A5/A6/A10/A11/A13/A14/A16/P in pancreatic cancer is positively correlated with TP53 mutation, while the high expression of S100A1/A12/B/Z is negatively correlated with TP53 mutation.

A correction has been made to the **Author Contributions**, sentence 2. The corrected sentence appears below:

XL, NQ, and QL performed critical revision of the manuscript.

In the Supplementary Material, there was a missing figure. A new Supplementary Figure 1 has been added, and the original Supplementary Figure 1 has been changed to Supplementary Figure 2.

The authors apologize for these errors and state that this does not change the scientific conclusions of the article in any way. The original article has been updated.

