# Corrigendum: Prognostic values and clinical significance of S100 family member’s individualized mRNA expression in pancreatic adenocarcinoma

**DOI:** 10.3389/fgene.2022.1057431

**Published:** 2022-10-21

**Authors:** Xiaomin Li, Ning Qiu, Qijuan Li

**Affiliations:** ^1^ Guangzhou Women and Children’s Medical Center, Guangzhou Medical University, Guangzhou, China; ^2^ Southern Marine Science & Engineering Laboratory (Guangzhou), Guangzhou, China; ^3^ Department of Clinical Laboratory, The Fifth Affiliated Hospital of Sun Yat-sen University, Zhuhai, China

**Keywords:** mRNA expression, S100 family, pancreatic cancer, biomarker, prognosis

In the published article, there was an error in affiliation 2. Instead of “Key Laboratory of Ocean and Marginal Sea Geology, Guangdong Southern Marine Science & Engineering Laboratory (Guangzhou), South China Sea Institute of Oceanology, Innovation Academy of South China Sea Ecology and Environmental Engineering, Chinese Academy of Sciences, Guangzhou, China”, it should be “Southern Marine Science & Engineering Laboratory (Guangzhou), Guangzhou, China”.

The authors apologize for this error and state that this does not change the scientific conclusions of the article in any way. The original article has been updated.

