# Erratum: Corrigendum: Prognostic values and clinical significance of S100 family member’s individualized mRNA expression in pancreatic adenocarcinoma

**DOI:** 10.3389/fgene.2022.1089027

**Published:** 2022-11-18

**Authors:** Frontiers Production Office

**Affiliations:** Frontiers Media SA, Lausanne, Switzerland

**Keywords:** mRNA expression, S100 family, pancreatic cancer, biomarker, prognosis

Due to a production error, the corrections requested in the Corrigendum were not implemented in the original article. Furthermore, the title of the original article was listed as “Pinocembrin Decreases Ventricular Fibrillation Susceptibility in a Rat Model of Depression,” but should have been “Prognostic Values and Clinical Significance of S100 Family Member’s Individualized mRNA Expression in Pancreatic Adenocarcinoma.”

The publisher apologizes for this mistake. The original version of this article, as well as the original article, have been updated.

