# Supplementary material for: Prognostic Values and Clinical Significance of S100 Family Member’s Individualized mRNA Expression in Pancreatic Adenocarcinoma
Source: Front Genet. 2021 Nov 3;12:758725. doi: 10.3389/fgene.2021.758725 (PMC8595214; doi:10.3389/fgene.2021.758725)
Supplement: Supplementary file 1 [file DataSheet1.doc]

Supplementary Material

# Supplementary Figures and Tables

## Supplementary Figures


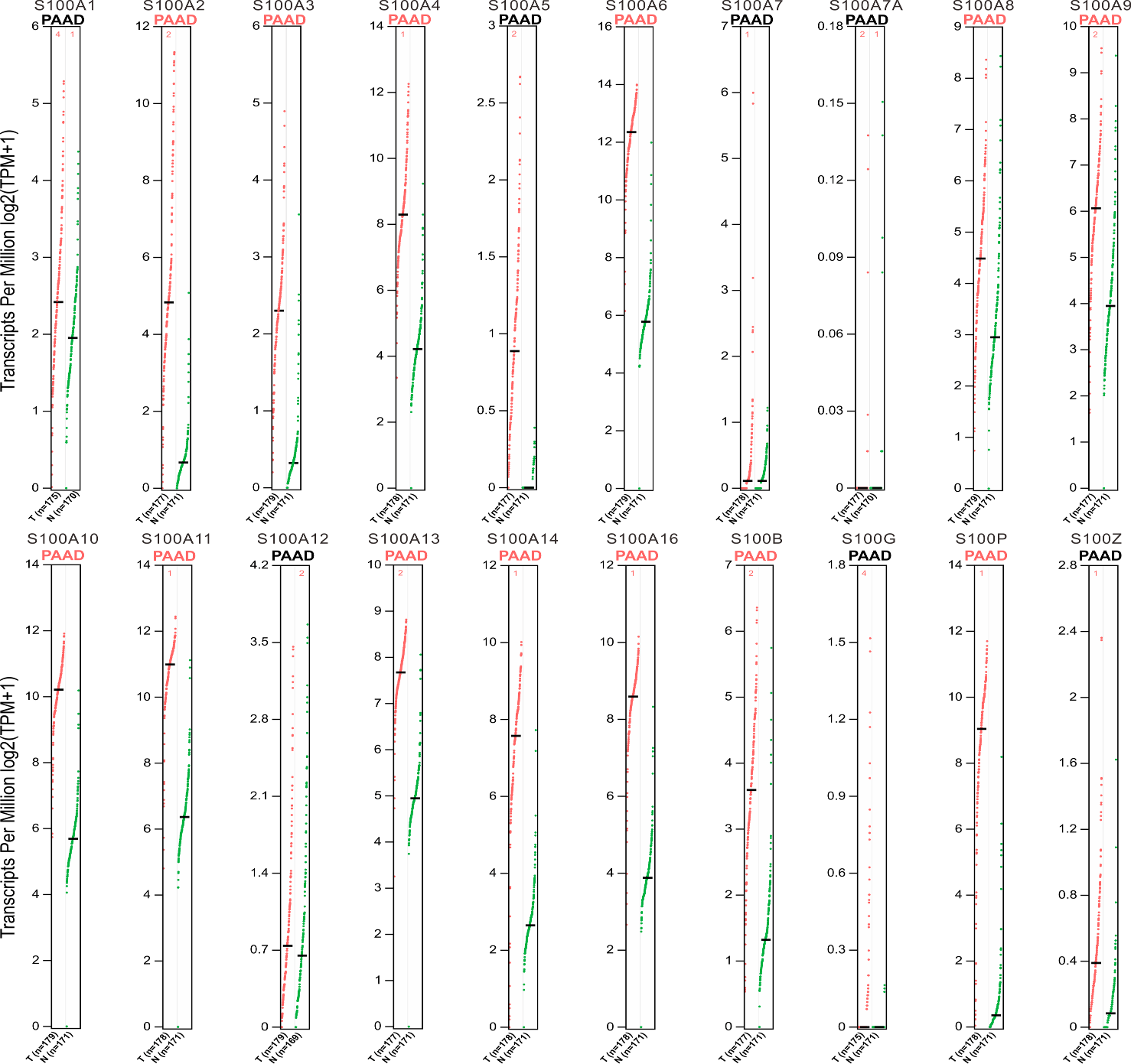


**Supplementary Figure 1.** Transcripts per million (TPM) of S100s mRNA in PAAD using GEPIA. Red color represents tumor tissue and green color represents normal tissue. PAAD marked in red indicates statistical difference, while PAAD marked in black indicates no difference.


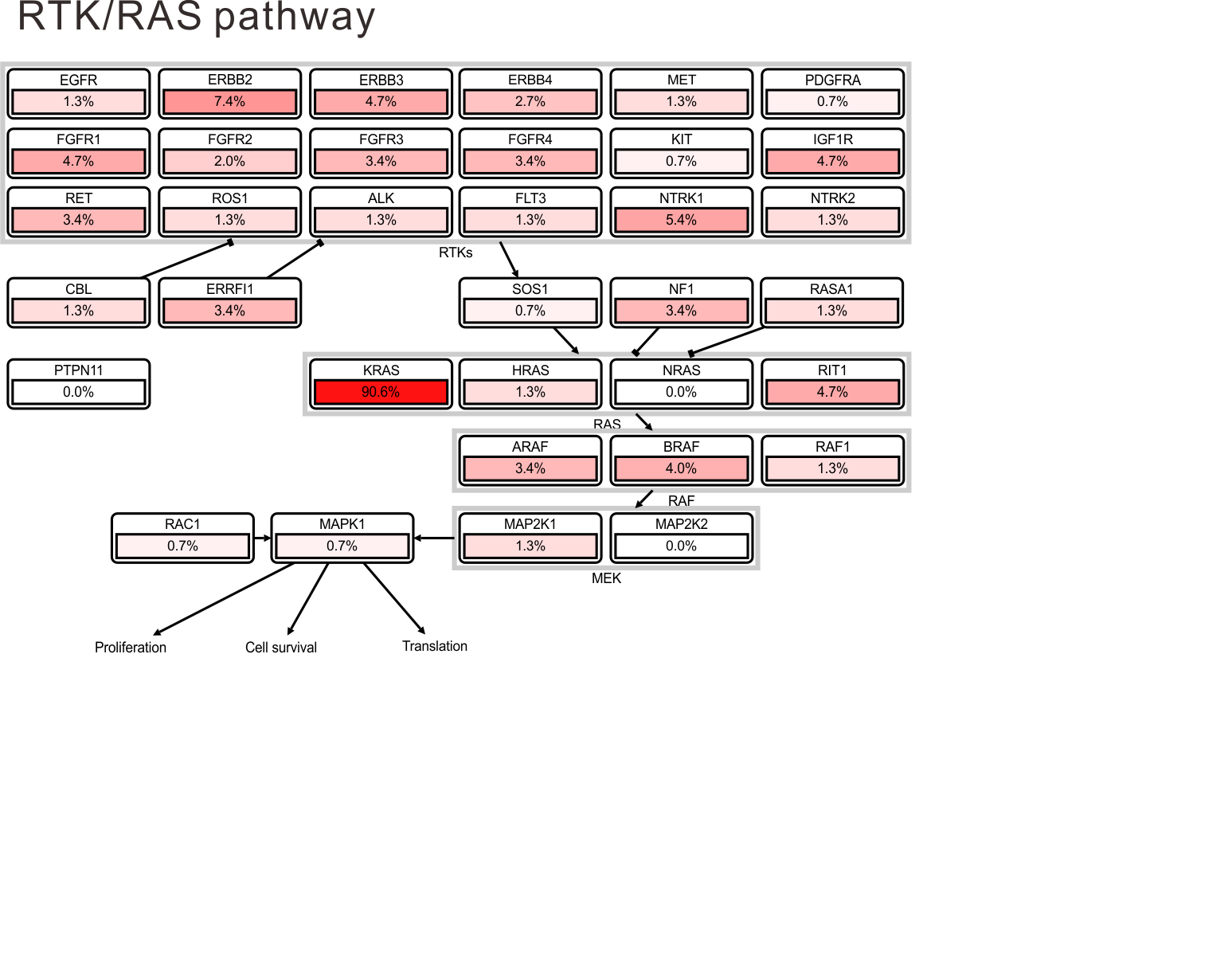


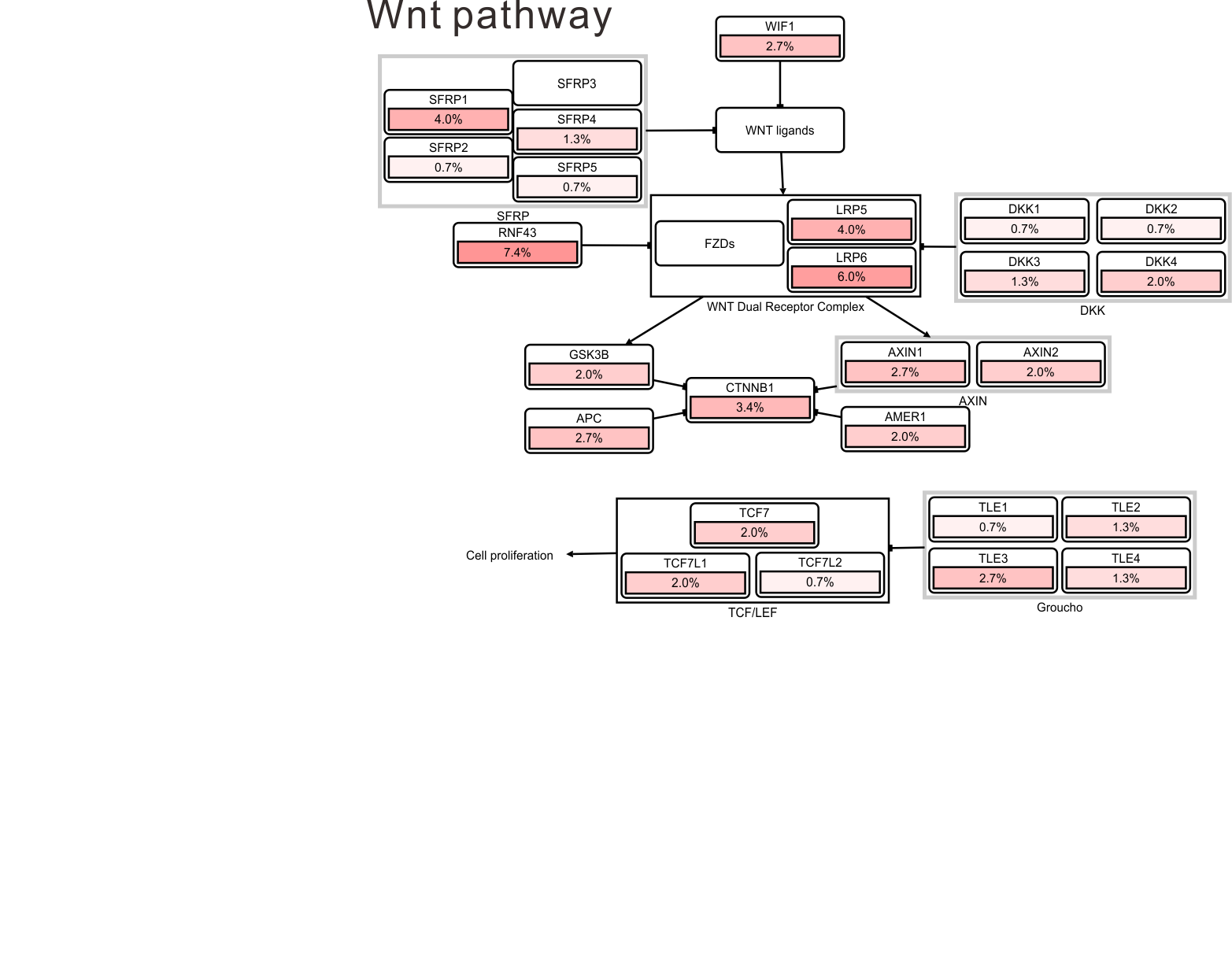


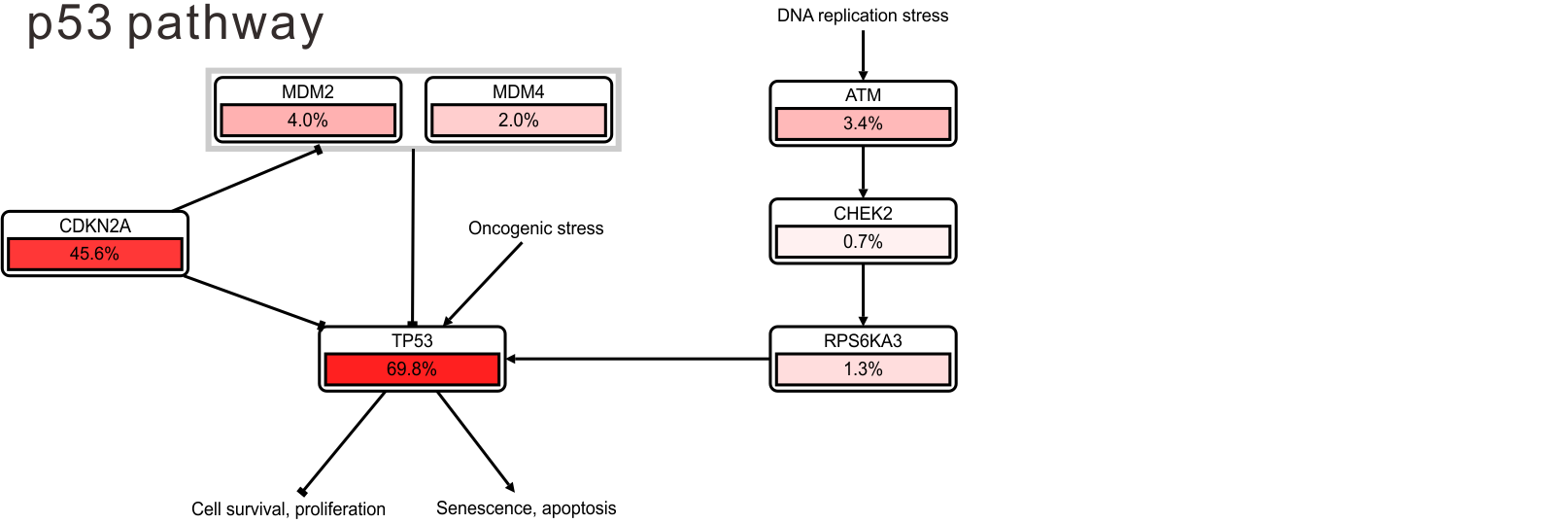


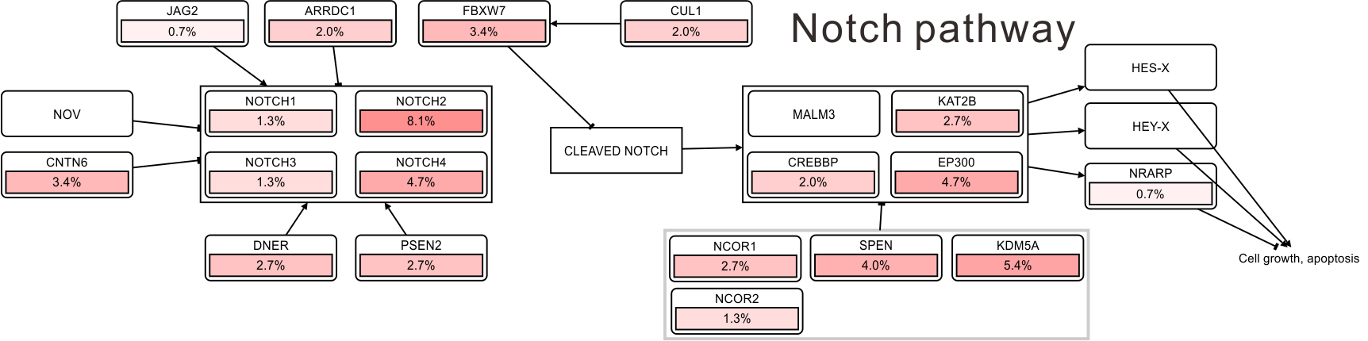


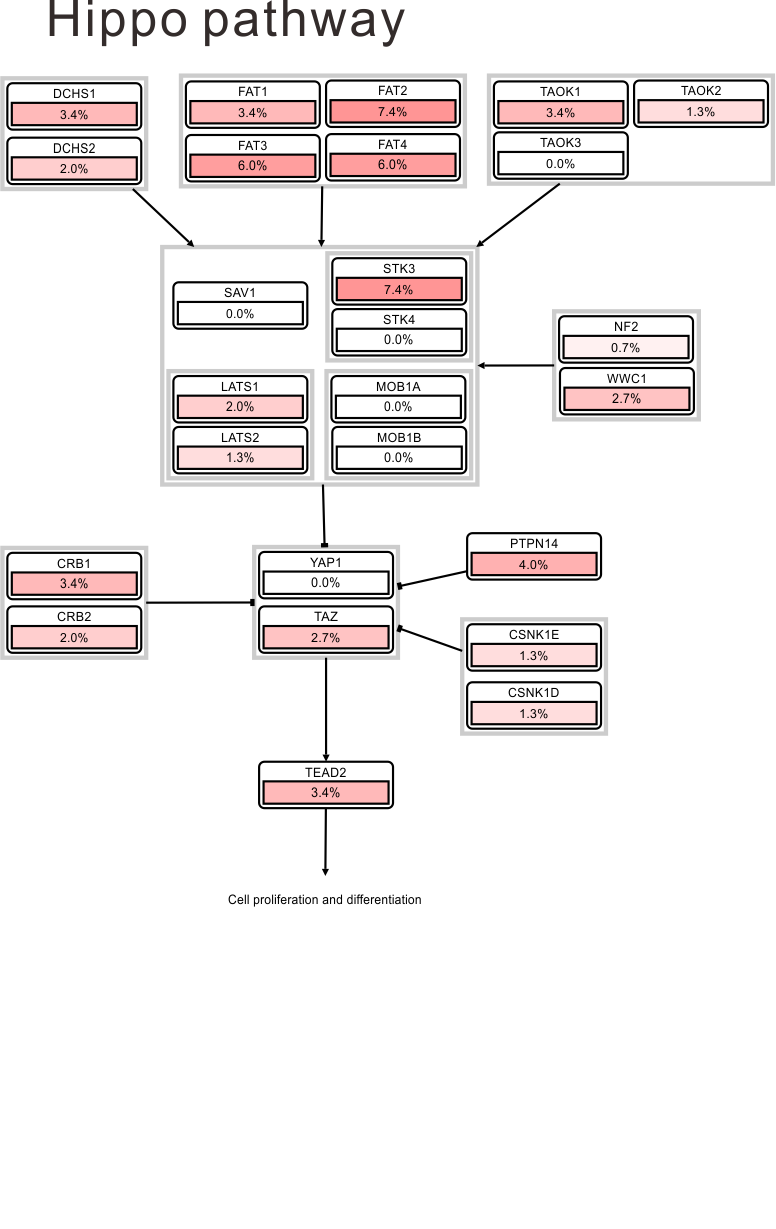


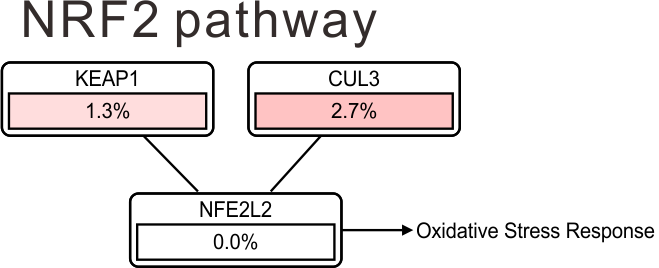

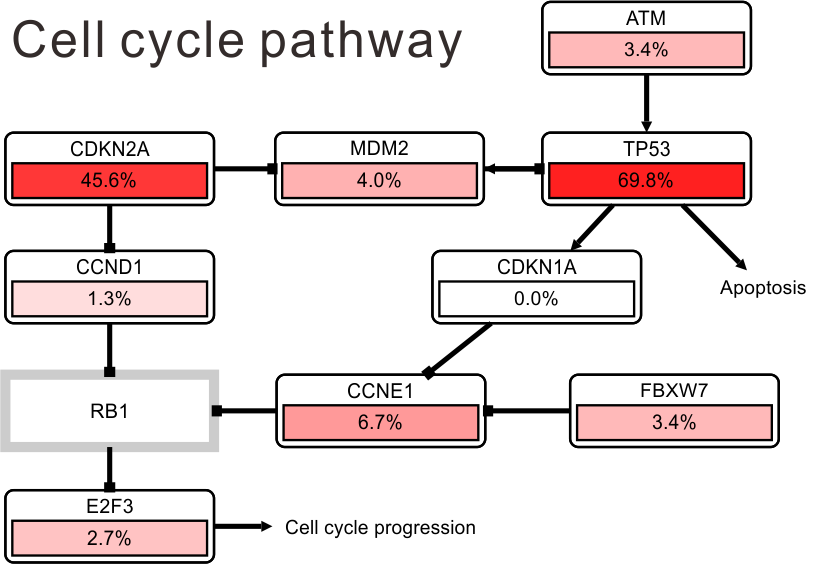


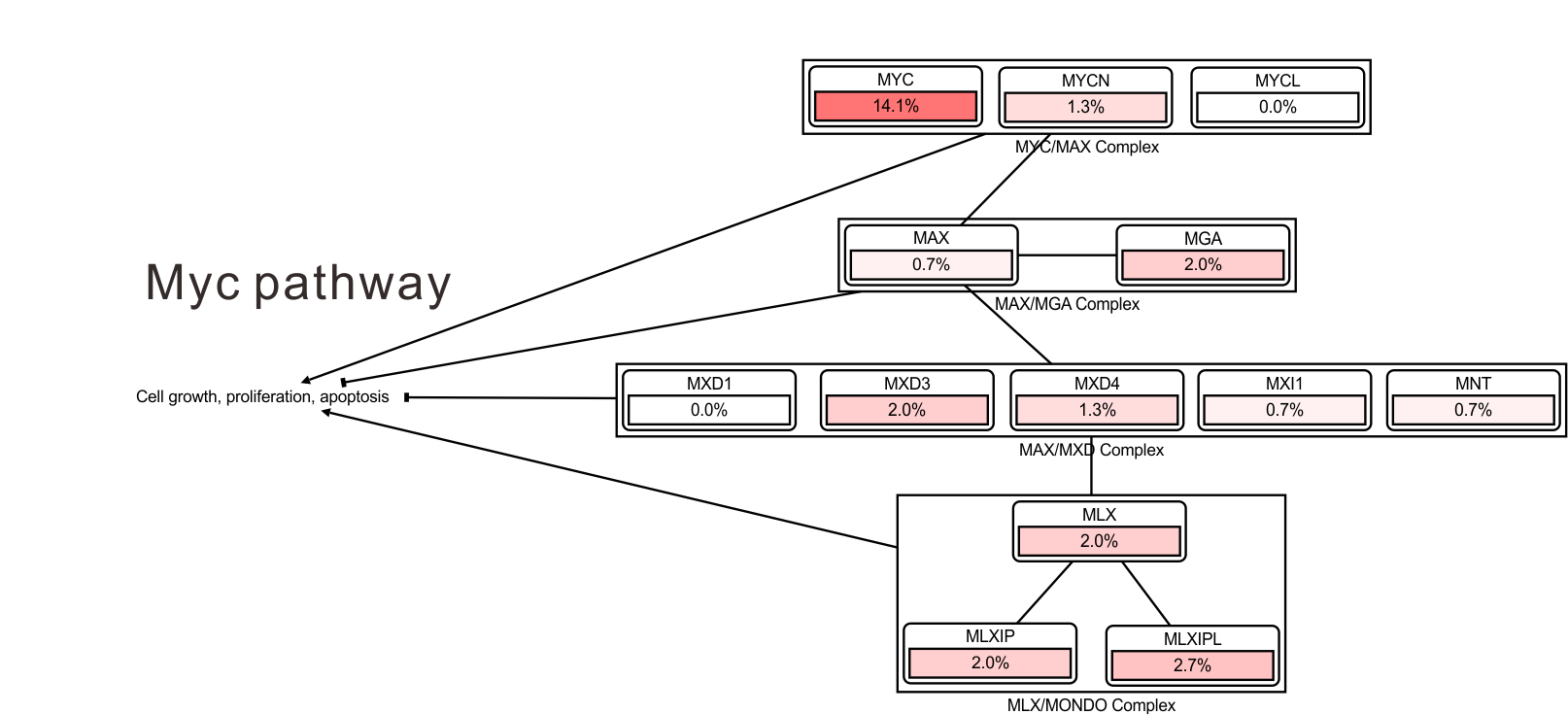


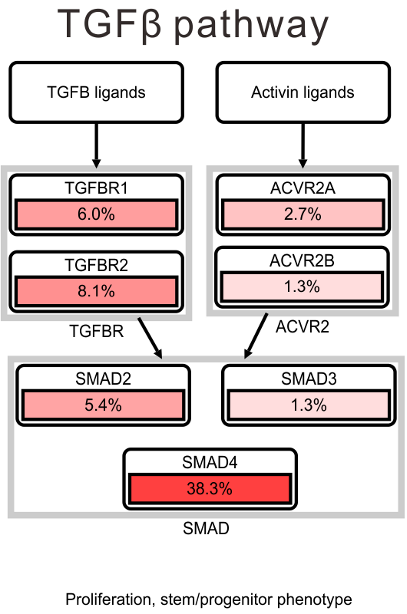

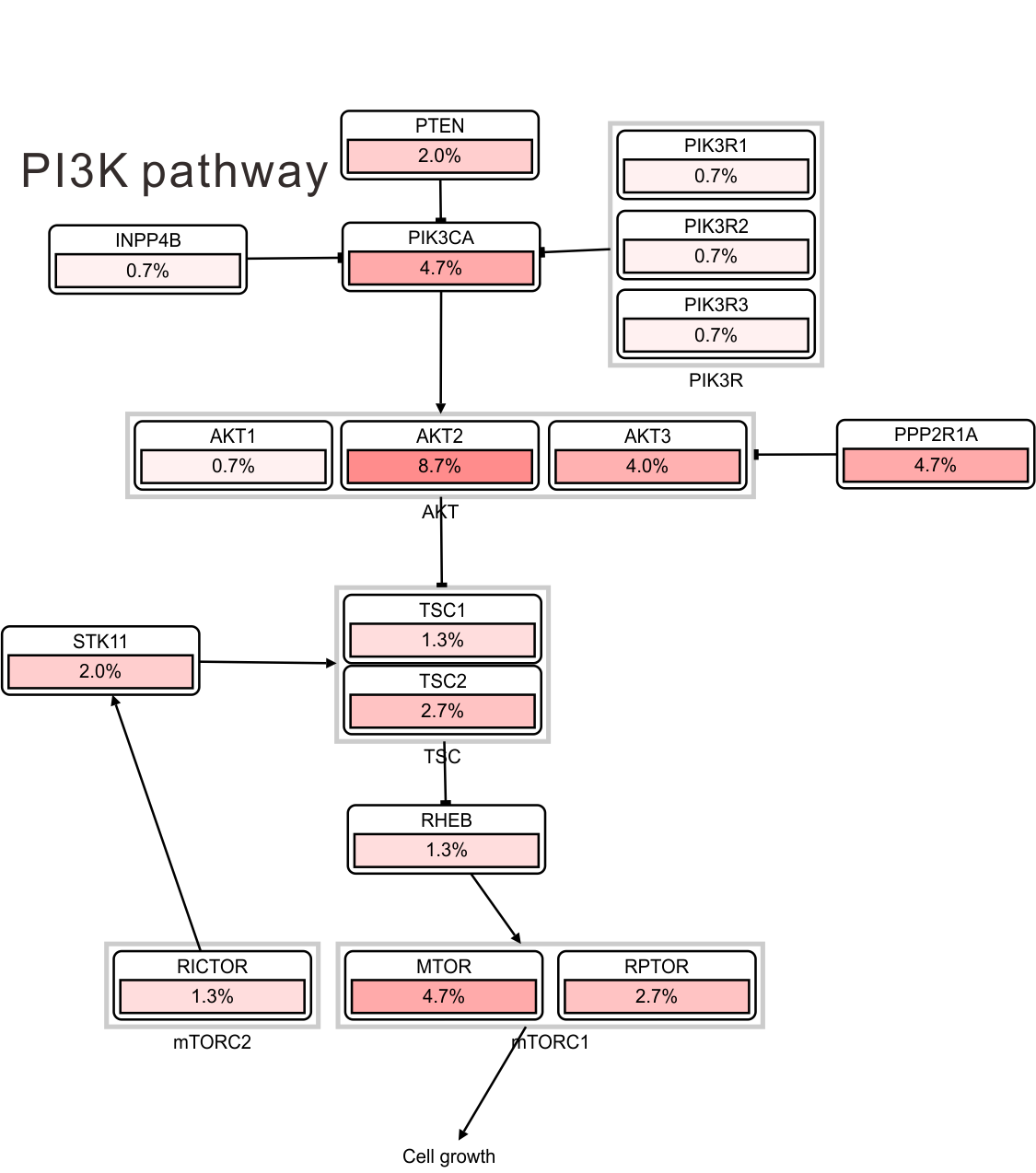


**Supplementary Figure 2**. Ten canonical oncogenic signaling pathways of the S100s in PAAD. Frequency of altered genes were indicated. Color intensity reflected the frequency of the alteration.
